# Regulation of a nickel tolerance operon conserved in *Mesorhizobium* strains from serpentine soils

**DOI:** 10.1128/aem.01403-25

**Published:** 2025-11-04

**Authors:** Kyson T. Jensen, Joel S. Griffitts

**Affiliations:** 1 Department of Microbiology and Molecular Biology, Brigham Young University 723033, Provo, Utah, USA; University of Illinois Urbana-Champaign, Urbana, Illinois, USA

**Keywords:** metalloregulation, CsoR/RcnR family

## Abstract

The *Mesorhizobium nre* (nickel resistance) operon has previously been shown to mediate Ni tolerance in serpentine soils with naturally high concentrations of this transition metal. In most serpentine-derived strains evaluated, the putative efflux genes *nreX* and *nreY* are conserved, along with a small gene (*nreA*) encoding a CsoR/RcnR-family transcriptional regulator. CsoR/RcnR-family regulators are small (around 100 amino acids in length); they bind transition metals; and they use an unconventional and poorly understood DNA-binding mechanism. NreA is 93 amino acids in length and belongs to a poorly characterized clade within the CsoR/RcnR family. This investigation is focused on regulatory DNA elements that functionally interact with *Mesorhizobium* NreA, as well as amino acid residues in NreA that influence its regulatory activity. We show that NreA is a transcriptional repressor that is responsive to exogenous Ni. The Ni-responsive promoter is immediately upstream of the *nreAXY* operon, resulting in a leaderless transcript. An operator sequence with dyad symmetry occupies the spacer region between the −35 and −10 promoter elements. Mutational analysis of this operator highlights functionally crucial base pairs occupying opposite ends of the 19 bp element. Changes to conserved residues in the NreA polypeptide result in varying effects, with changes predicted to prevent Ni binding leading to a super-repressor phenotype. Structural modeling of the NreA-operator complex provides a plausible mechanism for DNA binding by a tetrameric form of NreA, with DNA contact sites along a positively charged surface. The modeled contact sites agree well with the mutational analysis.

**IMPORTANCE:**

Bacteria employ diverse mechanisms for maintaining optimal intracellular levels of bioactive metals. Isolates of *Mesorhizobium* bacteria from Ni-rich serpentine soils possess a small genetic region controlling Ni efflux. This region is subject to transcriptional regulation via the small repressor protein NreA. In this work, the essential components of the NreA protein and the DNA operator with which it interacts are defined, enabling potential adaptation of this system for metal sensing or other technologies requiring a compact inducible gene expression module.

## INTRODUCTION

Bacteria have evolved diverse mechanisms to cope with metal stress, including efflux, sequestration, and detoxification of toxic metal ions (1–3). The fitness costs associated with these systems, as well as the need to precisely regulate the balance of intracellular metals, require bacteria to maintain precise control of metal stress responses (4). This control is primarily implemented through metal-responsive transcription factors or metalloregulators (5). Metalloregulators, which may be transcriptional activators or repressors, are generally homo-oligomeric proteins that interact with DNA-binding sites in upstream regulatory regions for genes controlling metal homeostasis. Metalloregulators also interact with specific metal ions that modulate binding to cognate DNA-binding sites (1, 2).

Repressors from the CsoR/RcnR metalloregulator family have received attention due to their small size and poorly defined mechanism of DNA binding (6–8). CsoR is a Cu-responsive regulator used by various gram-positive bacteria, and RcnR is a Ni- and Co-responsive regulator found in gram-negative enteric bacteria, including *Escherichia coli* (9, 10). Crystal structures of CsoR from *Mycobacterium tuberculosis* and *Streptococcus lividans* reveal a homodimeric or homotetrameric complex of alpha-helical bundles in which metal ions are coordinated in a cysteine- and histidine-containing coordination site formed at an intersubunit bridge (9, 11). A DNA-bound structure for this family has not been reported, though it has been presumed that a face of the tetrameric structure with strong positive electrostatic potential may mediate DNA binding (6, 8, 12, 13). Cu binding drives CsoR disengagement from operator DNA, and alterations to CsoR that prevent metal binding cause constitutive repression of the regulated operon (9, 14, 15). Metal coordination sites in CsoR and RcnR family members appear to be located at analogous intersubunit positions, though copper binding to CsoR is mediated by a Cys2His1 motif, while Ni/Co binding to RcnR is mediated by a Cys1His3 motif (9, 16).

Serpentine soils, defined by their unusual underlying geology, tend to be less hospitable to life because of stressful ionic imbalances and high levels of transition metals such as Cr, Co, and Ni (17). Ni levels in serpentine soils vary somewhat, ranging from 0.05 to 1.5 mM (18). A recent study of *Mesorhizobium* isolates from Ni-rich serpentine soils pointed us to a conserved Ni resistance operon containing a gene for a CsoR/RcnR-like protein that we termed NreA (18). The *nreA* gene is the most upstream in an operon that also encodes two independently functioning Ni efflux transporters: NreX and NreY (18). Here we characterize the mechanism by which NreA transcriptionally regulates the *nreAXY* operon.

## RESULTS

### Conserved sequences point to a minimal *nreAXY* promoter/operator

**Fig 1:**
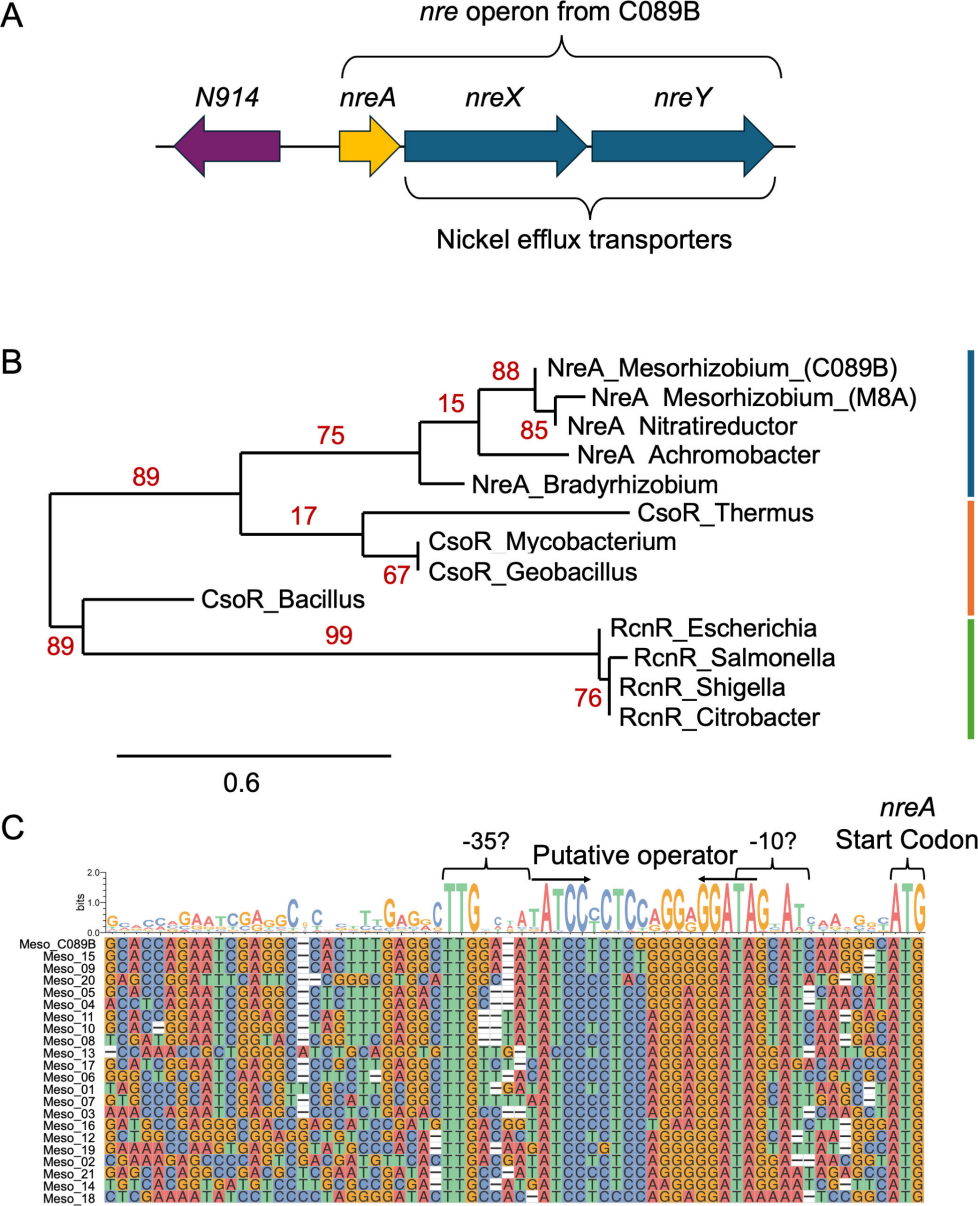
Comparison of *nreA* upstream regions points to probable regulatory elements. (**A**) Map of the *nre* operon identified in the Ni island of *Mesorhizobium* strain C089B. (**B**) Protein phylogenetic tree of members of the CsoR/RcnR family of transcriptional regulators. Support values are in red, and the scale bar indicates substitutions per site; NreA, CsoR, and RcnR clades are represented with blue, orange, and green lines, respectively. (**C**) Multiple sequence alignment of the *nre* upstream region from 21 different *Mesorhizobium* isolates, including C089B. Proposed promoter/operator elements are labeled, with arrows showing inverted repeats.

*Mesorhizobium* strain C089B is derived from a serpentine soil plot at McLaughlin natural reserve (California, USA) and contains an *nreAXY* operon that has previously been shown to mediate Ni tolerance (Fig. 1A [18]). The NreA polypeptide is similar in sequence to members of the CsoR/RcnR metalloregulator family, though it fits best into a phylogenetic clade with other uncharacterized NreA-like proteins from alpha- and beta-proteobacteria (Fig. 1B). In an initial attempt to discern a *cis*-acting regulatory region upstream of *nreA* that may be controlled by NreA, we compared sequences from 21 additional serpentine soil-derived *Mesorhizobium* strains (Fig. 1C). This DNA sequence alignment encompassed the *nreA* start codon on the right and approximately 65 bp upstream of the start codon. The sequence logo generated in Fig. 1C highlights a strongly conserved TTG motif most likely constituting the −35 RNA polymerase (RNAP) binding motif. A TAGNAT motif 20 bp to the right of this is likely a corresponding −10 RNAP binding motif. There is a strongly conserved, inverted-repeat sequence positioned between these putative −35 and −10 elements (the spacer region), which we presumed may function as an NreA operator. Notably, these elements occur quite close to the *nreA* start codon, suggesting they control transcription of an mRNA devoid of any substantial 5′ untranslated sequence.

**Fig 2:**
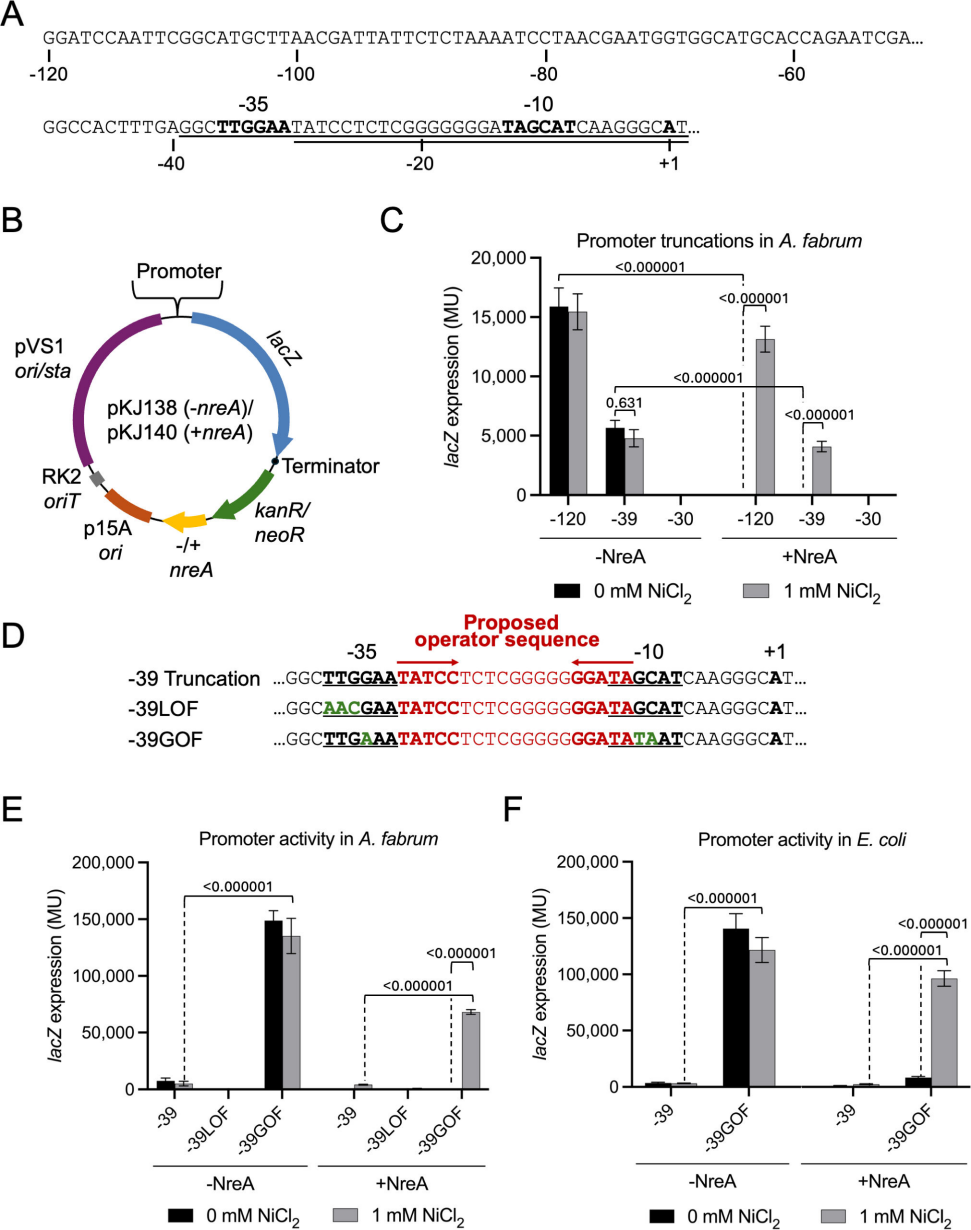
Identification of a minimal *nre* promoter and its functional elements. (**A**) Upstream sequence of the *nre* operon in strain C089B. Underlined sequences indicate 5′-truncated variants −39 and −30. Bolded characters represent a proposed −35 element, −10 element, and the translational start site. (**B**) Map of the binary reporter plasmid used to test promoter truncations and mutations in either *A. fabrum* or *E. coli*. pKJ138 is *nreA^−^*, while pKJ140 is *nreA^+^*. (**C**) Reporter gene activity from truncations of the *nre* promoter region. *A. fabrum* cells harboring *lacZ* fusions with or without *nreA* were grown in the presence or absence of supplemental NiCl_2_, and β-galactosidase activity was measured in Miller units (MU). Error bars represent standard deviation from the mean (*n* = 6). *P* values for *t*-tests of important comparisons are given. (**D**) Sequence alignment showing the −39 loss of function (LOF) and −39 gain of function (GOF) mutations (green). Underlined are putative −35 and −10 elements. Red denotes the putative operator sequence, and inverted arrows indicate the inverted repeats within this sequence. (**E and F**) Functional comparisons of promoter mutants shown in panel **D **were carried out in both *A. fabrum* (**E**) and *E. coli* (**F**). Data are formatted as in panel **C**. Error bars represent standard deviations from the mean (*n* = 6). *P* values for *t*-tests of important comparisons are given.

The conserved upstream sequence elements in the alignment in Fig. 1C set the stage for plasmid-based *lacZ* reporter analyses. We probed the functionality of a 120 bp upstream region or truncated variants thereof (Fig. 2A). Reporter assays were facilitated by a broad-host range shuttle vector (pKJ138, Fig. 2B) containing *lacZ* with a synthetic ribosome-binding site (RBS). This vector can accommodate the presence (pKJ140) or absence (pKJ138) of *nreA* downstream of the constitutively expressed kanamycin (Km) resistance gene. In this context, *nreA* possesses a strong RBS and is uncoupled from the transcriptional control region under investigation (see Fig. 2B). Most of the reporter gene tests described here were carried out in *Agrobacterium fabrum*, owing to its faster growth and easier genetic manipulation compared to wild *Mesorhizobium* strains. Using *A. fabrum*, we could also more confidently avoid potential cross-talk with co-evolving *Mesorhizobium* regulatory pathways.

When the full 120 bp region is tested in this system, we observe high-level expression that is repressed by *nreA* co-expression, and this *nreA*-dependent repression is alleviated by 1 mM NiCl_2_ (Fig. 2C). This Ni concentration was chosen because it is the highest concentration that allows both robust reporter gene expression and growth of *A. fabrum*. It is also within the range of Ni concentrations found in natural serpentine soils. We next tested whether truncation of the promoter region to the −39 position would still support Ni-inducible expression, supposing that maintenance of the core −35 and −10 promoter elements, as well as the putative operator, would allow regulated expression similar to the full-length 120 bp segment. The −39 construct supports reporter gene expression that is repressed by NreA and induced by Ni, but induced expression levels are lower by approximately threefold compared to the full-length construct (Fig. 2C). This may be explained by a positive regulatory element existing somewhere in the −120 to −39 region. However, given the robust inducible response we observed from the −39 construct, we proceeded to characterize the regulatory features within this much shorter sequence. As expected, the −30 construct did not support reporter gene expression under any condition, presumably due to loss of the −35 TTG element (Fig. 2C). In further support of the role of the −35 TTG element as a positive regulatory element, mutation of this sequence to AAC within the context of the −39 construct (−39 loss of function [LOF], Fig. 2D) yielded undetectable expression (Fig. 2E). On the other hand, a 3 bp change to the −39 construct designed to bring both the −35 and −10 elements closer to a prokaryotic promoter consensus sequence (−39 gain of function [GOF], Fig. 2D) led to greatly increased expression while maintaining the NreA-dependent, Ni-inducible property (Fig. 2E). Under inducing conditions, −39GOF supports approximately 16-fold higher expression, more than compensating for loss of the transcriptionally enhancing activity present in the −120 to −39 region. The −39GOF-*lacZ* construct also supports expression in the more distantly related *E. coli*, and this expression is also NreA repressed and Ni induced (Fig. 2F). In our attempt to enhance these positive regulatory elements, we intentionally avoided changes to the putative repressor-binding operator comprising the −30 to −12 region (red in Fig. 2D).

### The *nreAXY* operon is expressed as a leaderless mRNA

**Fig 3:**
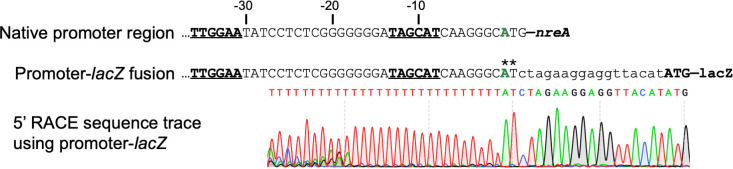
Identification of the *nre* transcription start site by 5′ rapid amplification of cDNA ends (5′-RACE). Analysis was carried out in *A. fabrum* carrying a promoter-*lacZ* fusion that includes the first two nucleotides of the native *nreA* start codon (**). Underlined are the −35 and −10 elements of the *nre* promoter. The first nucleotide of the detected transcript is shown in green. Bottom, inverted bottom-strand sequence trace acquired after poly-A tailing of the reverse-transcription product and subsequent PCR amplification.

The close proximity of the proposed promoter elements to the *nreA* start codon suggests that the transcript may be leaderless or may contain only a few untranslated nucleotides. Sequencing results from 5′ rapid amplification of cDNA ends (5′-RACE) support production of a leaderless transcript (Fig. 3). This test was carried out using an *A. fabrum* strain carrying the P*nreA-lacZ* −120 construct described above. This reporter fusion includes the first two base pairs of the native *nreA* start codon fused to a synthetic ribosome-binding site for efficient translation of the *lacZ* coding sequence, as illustrated in Fig. 3. In this context, the mRNA 5′ end mapped to the A nucleotide that corresponds to the native *nreA* AUG start codon. Unfortunately, difficulty in isolating quality RNA from *Mesorhizobium* cells prevented this test from being carried out in the strain naturally harboring the *nreAXY* operon. This finding defines the +1 site of transcription, as well as confirms the locations of the −10 and −35 promoter elements proposed above.

### Inverted repeats of the *nre* operator sequence are vital for repression

**Fig 4:**
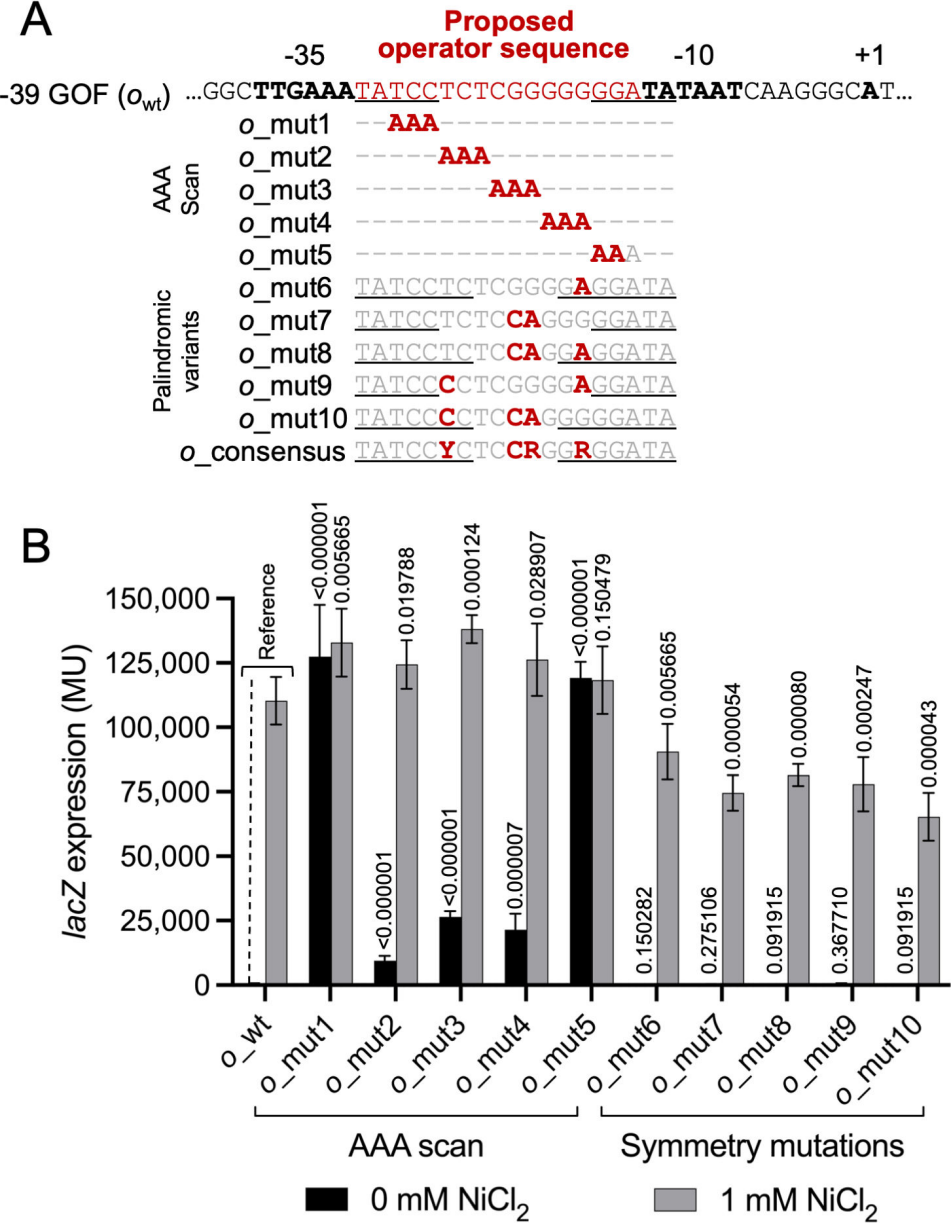
Identification of operator elements essential for NreA-mediated regulation. (**A**) Mutational analysis of the operator site. Black/bold: −35 and −10 elements; red/bold: mutations; black/underlined: inverted repeats. (**B**) Reporter gene activity resulting from mutations shown in panel **A**. *A. fabrum* cells (*nreA^+^*) harboring *lacZ* fusions were grown in the presence or absence of supplemental NiCl_2_, and β-galactosidase activity was measured in MU. Error bars represent the standard deviation from the mean (*n* = 6). *P* values are given based on comparisons with *o*_wt under identical growth conditions.

We next evaluated the role of the −30 to −12 spacer sequence, which we presumed to be the site of negative regulation by NreA. A set of mutant reporter fusions constituting a “scan” of AAA substitutions was tested (Fig. 4), and expression values indicate that the distal inverted repeats of this spacer (i.e., TCCN_9_GGA) are particularly crucial for negative regulation: both the TCC→AAA (*o*_mut1) and the GGA→AAA (*o*_mut5) substitutions result in fully constitutive reporter gene expression. The three AAA substitutions across the central N_9_ region (*o*_mut2 through *o*_mut4) resulted in more weakly constitutive phenotypes compared to the wild-type operator (*o*_wt_). In other words, these more central AAA mutants are mostly inducible (see Fig. 4B).

**Fig 5:**
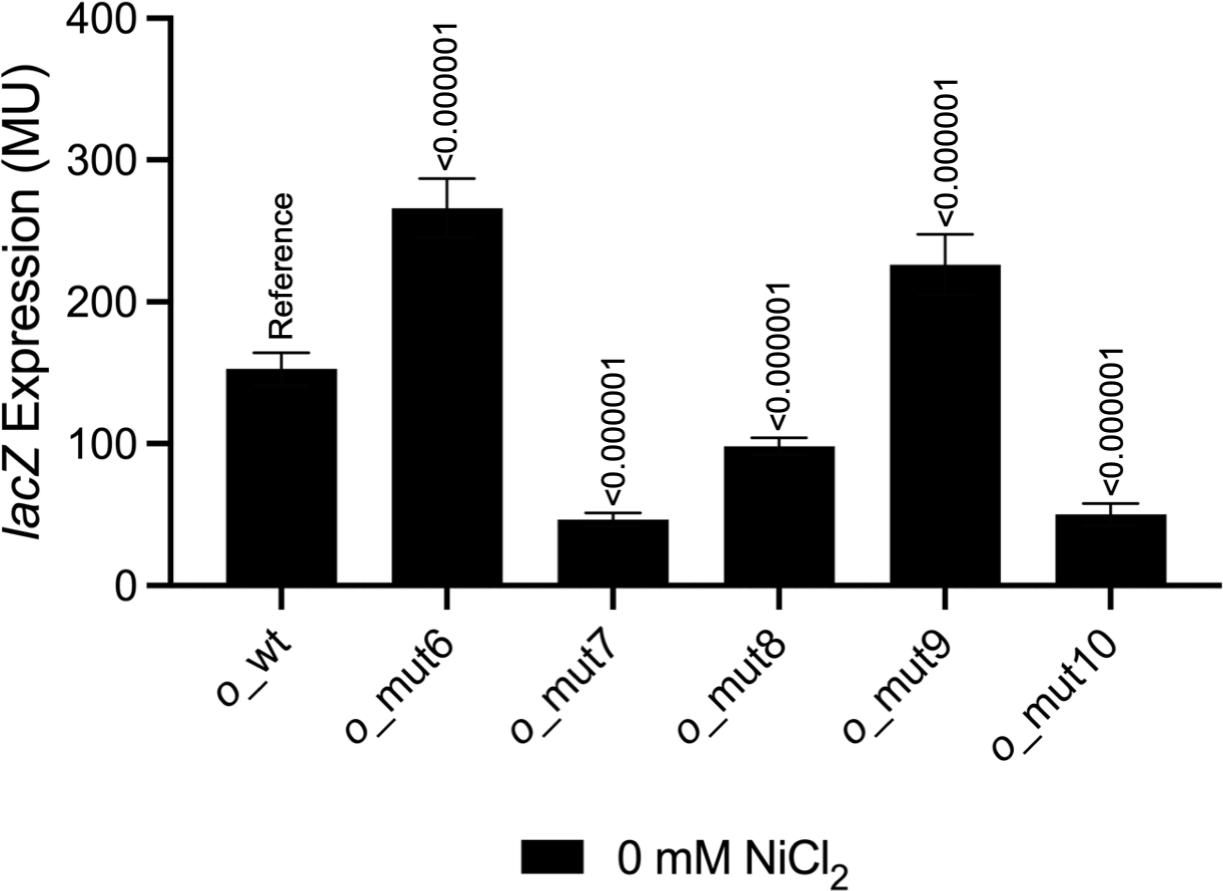
Comparison of expression levels from operator variants in medium without supplemental Ni. *A. fabrum* cells (*nreA*^+^) with *lacZ* fusions were measured for β-galactosidase activity (MU) after overnight culture and with an extended incubation time for the enzyme assay. Error bars represent standard deviation from the mean (*n* = 12). *P* values are given based on comparisons with *o*_wt under identical conditions.

The TCCN_9_GGA operator sequence from *Mesorhizobium* strain C089B (*o*_wt_) has dyad symmetry, and we know from results just presented that the distal ends of the inverted repeats are particularly crucial for operator function. The consensus sequence derived from an alignment of 22 *Mesorhizobium nre* promoter/operator regions (Fig. 1C) is more extensively symmetric, with the sequence TATCCYCtccrgGRGGATA, and some strains such as C089B contain a particularly G-rich sequence through the central region. It has been proposed for the metalloregulators RcnR and CsoR that a similar G-rich operator sequence may bring the DNA into an alternative hybrid A/B form to facilitate repressor binding (15, 19). In Fig. 4, operator variants *o_*mut6 through *o*_mut10 represent various alternative sequences that either increase the dyad symmetry of the central region or lessen its G-richness. In all cases, the variant operators support robust NreA-dependent repression and Ni inducibility. That one of these variants (*o*_mut8) has considerably less G-rich content and likely does not take on the alternative A/B hybrid DNA configuration suggests that NreA may be able to function on operators composed of ordinary B-form DNA. We investigated whether operators *o*_mut6 through *o*_mut10 might support even stronger repression than the native operator, *o*_wt_. We explored this low-range (leaky) expression by adding cells cultured overnight in the absence of Ni to our standard Miller assay and incubating the reaction for an extended period. Operators *o*_mut7 and *o*_mut10 exhibit three times more repression than *o*_wt_, with *o*_mut8 being intermediate in repression (Fig. 5). These three clones share the GG→CA substitution in the middle of the operator, suggesting that this specific change leads to stronger repressor binding. Operator variants *o*_mut6 and *o*_mut9, which do not contain the GG→CA substitution, are somewhat weaker in repression than *o*_wt_.

### Potential alternative NreA-responsive operators in the C089B genome

We carried out a search for other sequences in the *Mesorhizobium* C089B genome (NCBI accession number NZ_CP100469.1) that resemble the operator characterized above. As query sequences, we used both the native C089B *nreAXY* operator as well as the consensus operator derived from the alignment shown in Fig. 1C. We were able to identify several other candidate operator sequences (Table 3). One of the sequences is remarkably similar to the *nreAXY* operator and is located 2.07 Mb away, directly upstream of an *nreA*-like gene, which we named *nreA2*. For a comparison of these two sequences, see Fig. S1. Interestingly, *nreA2* is likely co-transcribed with an adjacent downstream gene encoding an NreX-like transport protein (*nreX2*) (Fig. S1). Based on amino acid sequence alignments, NreA and NreA2 are 55% identical, and NreX and NreX2 are 68% identical. It is therefore possible that these two operons function with some redundancy, although we have previously shown that the *nreXY* deletion in C089B confers considerable loss of Ni tolerance (18).

### Conserved NreA residues influence either DNA-binding or metal-binding activities

**Fig 6:**
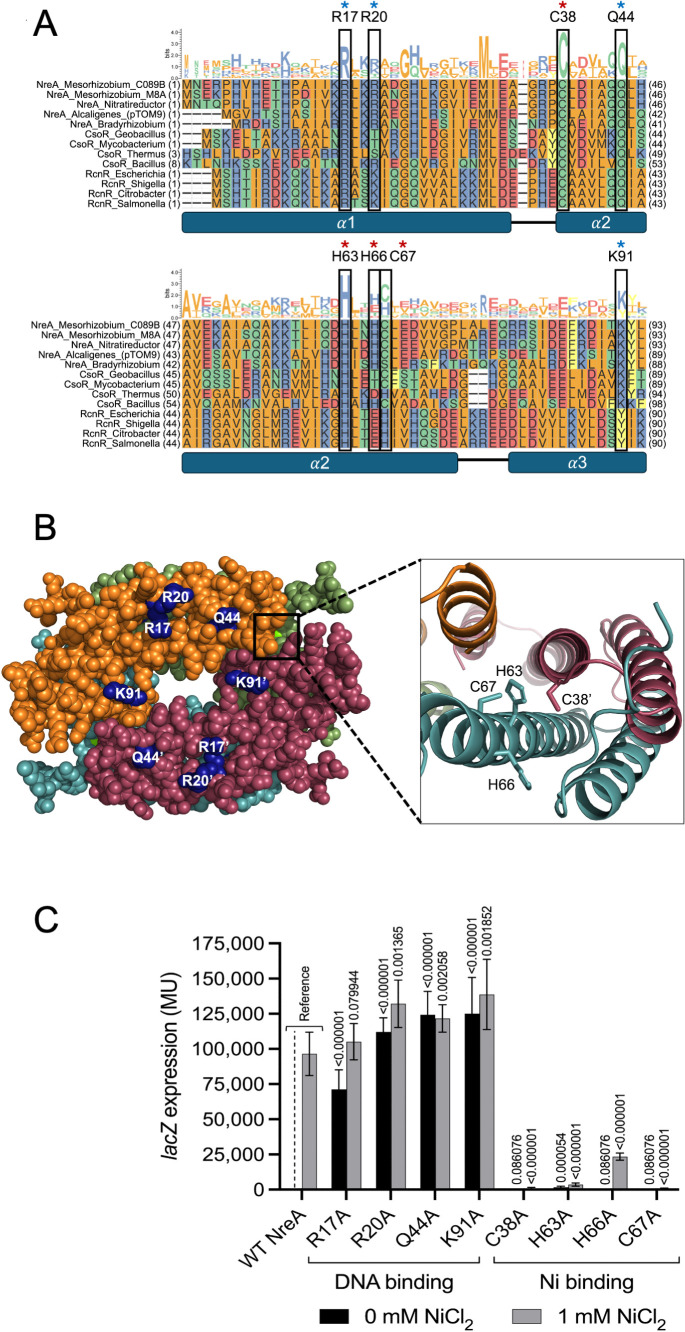
Influence of conserved NreA residues on NreA-mediated transcriptional regulation. (**A**) Protein alignment of NreA and CsoR/RcnR family members from diverse bacteria. Boxes indicate conserved residues that were individually altered in this experiment. Blue asterisks denote residues thought to be involved in DNA binding, and red asterisks denote residues implicated in metal binding. (**B**) AlphaFold 3 prediction of the metal-bound form of NreA with proposed conserved DNA-interacting residues highlighted in blue. The different subunits of the NreA tetramer are colored (orange, red, green, and cyan). One of the four metal ions in this model is partially visible and indicated with a box. In the close-in view of the modeled metal-binding pocket, the ion has been removed from this rendering for clarity. (**C**) Reporter gene activity resulting from amino acid substitutions of NreA. *A. fabrum* cells harboring *lacZ* fusions to the −39GOF promoter/operator were grown in the presence or absence of supplemental NiCl_2_. β-Galactosidase activity was measured in MU. Error bars represent standard deviations from the mean (*n* = 6). *P* values are given based on comparisons with wild-type NreA under identical conditions.

The *Mesorhizobium* NreA protein bears extensive sequence similarity to CsoR/RcnR family members (Fig. 6A). We generated a structural model of an NreA tetramer (Fig. 6B), and this model corresponds well with a representative crystal structure of CsoR from *Thermus thermophilus* HB8 (PDB 3AAI, root mean square [RMS] score of 1.443). The amino acids best conserved in the sequence alignment generally correspond either to an intersubunit metal-binding pocket (NreA residues C38, H63, H66, and C67) or to a potential DNA-binding surface (NreA residues R17, R20, Q44, and K91). We performed inducibility tests for strains in which each of these conserved NreA residues was substituted with alanine. In Fig. 6C, we see that alterations across the putative DNA-binding surface yield mostly or completely constitutive phenotypes, consistent with loss of operator binding. Substitutions around the putative Ni-binding pocket, on the other hand, give rise to mostly or completely super-repressing phenotypes; that is, repression persists even in the presence of Ni. The partial functionality of the H66A variant is consistent with the structural model (Fig. 6B), which places this residue at a location peripheral to the binding pocket. On the other hand, the alanine substitutions to more interior Cys/His residues (C38A, H63A, and C67A) result in strong repression that is no longer relieved by exogenous Ni.

## DISCUSSION

We have characterized the transcriptional control of a metal-responsive operon (*nreAXY*) in *Mesorhizobium* initially discovered for its role in evolutionary adaptation to Ni-rich serpentine soils (18, 20). Using phylogenetic analysis coupled with a heterologous *Agrobacterium*-based reporter system, we have identified NreA (a CsoR/RcnR family member) as a Ni-responsive transcriptional repressor, as well as *cis*-acting DNA sequences crucial to metalloregulation of this operon. We have further implicated specific residues in the NreA repressor as being important for either DNA binding or metal-mediated transcriptional induction.

Naturally diverse alleles of the *nre* operon in *Mesorhizobium* allowed us to pinpoint *cis*-acting regulatory sequences under strong selective pressure, and we mapped this *cis*-regulatory region to a remarkably compact segment of DNA comprising the 39 bp upstream of the *nreA* start codon. An operator sequence with dyad symmetry positioned within the −35/−10 spacer segment mediates repression by NreA. Operators interacting with CsoR/RcnR repressors are divided into two general types: type 1 operators consist of a central GC-tract flanked by inverted repeats, while type 2 operators consist of a longer central region with two or more G/C tracts flanked by inverted repeats. In some CsoR/RcnR-controlled promoters, two operator sequences (of either type) may be situated in close proximity with each other (15, 19). The NreA operator (TATCCtctcgggggGGATA) is type 1, with a single G_7_ tract. The inverted repeats flanking the central G-rich region are critical for NreA-mediated repression (Fig. 4). Mutations to the G-rich central region result in more subtle de-repression phenotypes. However, operator variants designed to increase dyad symmetry or to reduce G-richness while maintaining the consensus sequence retained repression, with some yielding stronger repression than the wild-type operator (Fig. 5). These findings suggest that NreA may function independently of DNA structural features such as A/B-form hybrids, which have been proposed to facilitate RcnR binding to G-rich operators (10, 15, 19). Indeed, NreA-interacting operator variants with a central GG→CA mutation showed enhanced repression, and a more severe GGG→AAA mutation (Fig. 4, *o*_mut4) retains some operator activity, indicating that specific base pair contacts, rather than overall helical structure, may dominate NreA-DNA interactions (see Fig. S2). Discerning sequence-specific and structure-mediated modes of operator-NreA interaction would require further investigation.

Like other CsoR/RcnR family members, NreA monomers are small (93 aa in length). While proteins in this family form alpha-helical bundles as monomers, they tend to form higher-order tetrameric structures (dimer of dimers) (9, 10, 15, 19, 21, 22). A structural model for NreA in a metal-bound state was generated, and this model places a metal-binding pocket with highly conserved His and Cys residues at intersubunit junctions (Fig. 6C). Alanine substitution of the putative metal-coordinating residues (C38, H63, and C67) resulted in a super-repressed state in our reporter assay system, consistent with failure to respond to the Ni inducer (Fig. 6D). None of these metal-binding pocket substitutions mimicked a metal-bound (promoter-ON) phenotype; this may be attributed to the NreA tetramer being in a relaxed, DNA-binding state in the apo form, with metal binding introducing tension and DNA release. Substitutions containing side chains that are more bulky or positively charged may lead to metal-mimicking, non-repressing NreA variants. Only the tetrameric (or larger) configuration of NreA forms a modeled structure with the potential to bind metals and the operator of the sequence we have determined in this work. Computational docking of the operator to the NreA tetramer confirms a generally good fit to a positively charged surface on the tetramer (Fig. S2). Using structure-guided substitutions, we show that NreA-mediated repression is dependent on a set of conserved residues likely located on this DNA-binding surface (Fig. 6B and C).

While considerable polymorphism exists among *nre*-containing *Mesorhizobium* strains—even in the *nreAXY* promoter region—they appear to uniformly produce a leaderless mRNA transcript devoid of a Shine-Dalgarno (SD) translation initiation enhancer, suggesting an adaptive advantage associated with this unusual mRNA structure. An apparent association between leaderless transcription and stress responses has previously been discussed (23, 24). When leaderless mRNAs engage directly with 70S ribosomes, certain time- and energy-consuming steps associated with conventional SD-mediated translation initiation are bypassed; in stressful circumstances, this enhanced economy may support more rapid stress-response protein expression.

From an applied perspective, the NreA-controlled promoter/operator, with some engineering, is functional in *A. fabrum* and *E. coli*, demonstrating its broad portability and independence from species-specific factors. The full regulatory module (promoter/operator and repressor-coding sequence) can be encoded on as little as 330 bp of DNA while enabling strongly inducible transgene expression. This genetic module is amenable to engineering for tuning the strength of repression or induction and possibly shifting metal specificity. Such a module could be adapted for use as a biosensor of various metals of biological and environmental importance. For example, the −39GOF version of the *nreA* promoter responds to exogenous Ni as low as 0.0625 mM (Fig. S3). Super-repressor variants of NreA may be useful as compact DNA-binding reagents for applications such as *in vivo* locus tracking or plasmid display.

## MATERIALS AND METHODS

### Bacterial strains and culture conditions

Strains used were *Mesorhizobium* C089B (18), *Agrobacterium fabrum* D224 (a streptomycin [Sm]-resistant variant of UBAPF2) (25), *Escherichia coli* DH5α (26), and DH5α-based conjugation helper strain B001 (27). Cells were cultured in Luria-Bertani (LB) broth (10 g/L tryptone, 5 g/L yeast extract, and 5 g/L NaCl_2_). When appropriate, 12 g/L of agar was added as a solidifying agent. Media supplements and antibiotics were as follows: Sm (200 µg/mL), Km (30 µg/mL), neomycin (Nm, 100 µg/mL), chloramphenicol (30 µg/mL), and nickel(II) chloride (1 mM). *Mesorhizobium* and *Agrobacterium* strains were grown at 30°C, and *E. coli* strains were grown at 37°C. Triparental matings were carried out at 30°C. All engineered strains used in this study are listed in Table S1 in the Supplemental Materials.

### Plasmid and strain construction

For testing promoter/operator constructs in *A. fabrum*, *lacZ* reporter plasmids pKJ138 (*nreA*−) and pKJ140 (*nreA*+) were used (Fig. 2B). These plasmids include a p15A origin for replication in *E. coli* and a pVS1 *ori/sta* cassette for replication and stability in *A. fabrum*. The RK2 *oriT* is included to make the plasmids mobilizable, and the *E. coli lacZ* reporter gene is appended with a strong synthetic RBS. For pKJ140, *nreA* is cloned with a synthetic RBS immediately downstream of the Km/Nm resistance gene, and expression of this bicistronic operon is presumed to be constitutive. Complete annotated sequences for pKJ138 and pKJ140 are given in the Supplemental Materials. Promoter/operator variants (assembled as hybridized oligonucleotides) were ligated upstream of *lacZ* using BamHI and XbaI sites (Table 1). For creating NreA alanine substitution variants (Table 2), the wild-type *nreA* gene was first cloned in the small plasmid, pKJ213 (Fig. S4, sequence in the Supplemental Materials), with which mutagenesis oligonucleotides were used to incorporate changes via inverse-PCR followed by circularization with T4 DNA ligase and T4 polynucleotide kinase. The resulting *nreA* variants were then amplified with primers oKJ614 and oKJ615 and inserted into pKJ227 (pKJ138 with the −39GOF promoter/operator), via a KpnI site. All oligonucleotide sequences used for producing promoter/operator and *nreA* variants are given in Table S2 in the Supplemental Materials. All plasmids used in this study were verified by Sanger sequencing. Reporter plasmids were transferred to *A. fabrum* D224 via triparental mating using the helper strain (B001) and donor strain (DH5α) containing the experimental plasmids. For conjugation, strain mixtures were co-cultured on LB agar at 30°C for 4–24 h and plated on LB agar containing Sm and Nm.

**TABLE 1:** Variant promoter/operator sequences used in this study

Variant	Test plasmid(s)^*a*^	Sequence^*b*^	Oligonucleotides
−120 truncation	pKJ217/218	ggatccAATTCGGCATGCTTAACGATTATTCTCTAAAATCCTAACGAATGGTGGCATGCACCAGAATCGAGGCCACTTTGAGGCTTGGAATATCCTCTCGGGGGGGATAGCATCAAGGGCAtctaga	oKJ527/564 (PCR)
−39 truncation	pKJ219/220	ggatccGGCTTGGAATATCCTCTCGGGGGGGATAGCATCAAGGGCAtctaga	oKJ498/499
−30 truncation	pKJ221/222	ggatccTATCCTCTCGGGGGGGATAGCATCAAGGGCAtctaga	oKJ575/576
−39LOF	pKJ225/226	ggatccGGCaacGAATATCCTCTCGGGGGGGATAGCATCAAGGGCAtctaga	oKJ502/503
−39GOF	pKJ227/228	ggatccGGCTTGaAATATCCTCTCGGGGGGGATAtaATCAAGGGCAtctaga	oKJ504/505
*o*_mut1	pKJ238	ggatccGGCTTGaAATA**aaa**TCTCGGGGGGGATAtaATCAAGGGCAtctaga	oKJ554/555
*o*_mut2	pKJ240	ggatccGGCTTGaAATATCC**aaa**CGGGGGGGATAtaATCAAGGGCAtctaga	oKJ556/557
*o*_mut3	pKJ242	ggatccGGCTTGaAATATCCTCT**aaa**GGGGGATAtaATCAAGGGCAtctaga	oKJ558/559
*o*_mut4	pKJ244	ggatccGGCTTGaAATATCCTCTCGG**aaa**GGATAtaATCAAGGGCAtctaga	oKJ560/561
*o*_mut5	pKJ246	ggatccGGCTTGaAATATCCTCTCGGGGG**aa**ATAtaATCAAGGGCAtctaga	oKJ562/563
*o*_mut6	pKJ231	ggatccGGCTTG**a**AATATCCTCTCGGGG**a**GGATAtaATCAAGGGCAtctaga	oKJ565/566
*o*_mut7	pKJ232	ggatccGGCTTG**a**AATATCCTCTC**ca**GGGGGATAtaATCAAGGGCAtctaga	oKJ567/568
*o*_mut8	pKJ233	ggatccGGCTTG**a**AATATCCTCTC**ca**GGaGGATAtaATCAAGGGCAtctaga	oKJ569/570
*o*_mut9	pKJ234	ggatccGGCTTG**a**AATATCC**c**CTCGGGG**a**GGATAtaATCAAGGGCAtctaga	oKJ571/572
*o*_mut10	pKJ235	ggatccGGCTTG**a**AATATCC**c**CTC**ca**GGGGGATAtaATCAAGGGCAtctaga	oKJ573/574

^*a*^ Plasmids are compatible for use in *A. fabrum* or *E. coli*. Strains harboring these plasmids are listed in Table S1. Where two plasmids are listed, the first is *nreA*^−^, and the second is *nreA*^+^. If only one plasmid is listed, it is *nreA*^+^.

^*b*^ Sequence includes the BamHI (GGATCC) and XbaI (TCTAGA) sites used for ligating into pKJ138 (*nreA^−^*) and/or pKJ140 (*nreA^+^*). Lowercase letters within the sequence represent cloning sites. Bold lowercase letters represent variations compared to the −39 truncation clone. For empty vectors pKJ138 and pKJ140, the BamHI/XbaI region contains the following sequence: ggatccACCtctaga.

**TABLE 2:** NreA variants tested in *A. fabrum*

Variant	Mutagenesis plasmid	Test plasmid	Sequence^*a*^	Mutagenesis oligonucleotides^*b*^
C089B NreA (wild type)	pKJ213	pKJ257	MNERPHVHETHPAIIKRLKRADGHLRGIVEMIEAGRPCLDIAQQLHAVEKAIAQAKKTLIQDHLNHCLEDVVGPLALEQRRSIDEFKDITKYL	oKJ608/609
R17A	pKJ216	pKJ260	MNERPHVHETHPAIIK**a**LKRADGHLRGIVEMIEAGRPCLDIAQQLHAVEKAIAQAKKTLIQDHLNHCLEDVVGPLALEQRRSIDEFKDITKYL	oKJ585/586
R20A	pKJ255	pKJ267	MNERPHVHETHPAIIKRLK**a**ADGHLRGIVEMIEAGRPCLDIAQQLHAVEKAIAQAKKTLIQDHLNHCLEDVVGPLALEQRRSIDEFKDITKYL	oKJ610/611
C38A	pKJ270	pKJ258	MNERPHVHETHPAIIKRLKRADGHLRGIVEMIEAGRP**a**LDIAQQLHAVEKAIAQAKKTLIQDHLNHCLEDVVGPLALEQRRSIDEFKDITKYL	oKJ581/582
Q44A	pKJ252	pKJ264	MNERPHVHETHPAIIKRLKRADGHLRGIVEMIEAGRPCLDIAQ**a**LHAVEKAIAQAKKTLIQDHLNHCLEDVVGPLALEQRRSIDEFKDITKYL	oKJ604/605
H63A	pKJ215	pKJ259	MNERPHVHETHPAIIKRLKRADGHLRGIVEMIEAGRPCLDIAQQLHAVEKAIAQAKKTLIQD**a**LNHCLEDVVGPLALEQRRSIDEFKDITKYL	oKJ583/584
H66A	pKJ247	pKJ261	MNERPHVHETHPAIIKRLKRADGHLRGIVEMIEAGRPCLDIAQQLHAVEKAIAQAKKTLIQDHLN**a**CLEDVVGPLALEQRRSIDEFKDITKYL	oKJ596/597
C67A	pKJ253	pKJ265	MNERPHVHETHPAIIKRLKRADGHLRGIVEMIEAGRPCLDIAQQLHAVEKAIAQAKKTLIQDHLNH**a**LEDVVGPLALEQRRSIDEFKDITKYL	oKJ606/607
K91A	pKJ256	pKJ268	MNERPHVHETHPAIIKRLKRADGHLRGIVEMIEAGRPCLDIAQQLHAVEKAIAQAKKTLIQDHLNHCLEDVVGPLALEQRRSIDEFKDIT**a**YL	oKJ612/613

^*a*^ Sequence includes the entire NreA amino acid sequence for NreA variants used. Bold lowercase letters represent alanine substitutions compared to the C089B NreA (wild type).

^*b*^ Mutagenesis oligonucleotides were used to incorporate mutations in *nreA* via inverse-PCR amplification of pKJ213, followed by circularization, followed by transfer of the variant gene to pKJ227, as described in Materials and Methods. *A. fabrum* strains harboring these plasmids are listed in Table S1.

### *lacZ* expression assays

Modified Miller assays similar to Zhang and Bremer (28) were carried out as follows: bacteria were grown in liquid culture overnight and their absorbance at 600 nm (*A*_600_) was measured; culture density was then adjusted to an *A*_600_ value of 0.2 using LB broth. These suspensions were diluted 10-fold into 200 μL of LB in 96-well plates and cultured with shaking (~300 rpm) at 30°C for 6 h, with half-strength antibiotics (Sm-100 and Nm-50). From these cultures, 2 μL of suspension was transferred to 23 µL of permeabilization solution (100 mM Na_2_HPO_4_, 20 mM KCl, 2 mM MgSO_4_, 0.8 mg/mL hexadecyltrimethylammonium bromide, 0.4 mg/mL sodium deoxycholate, and 5.4 µL/mL beta-mercaptoethanol), incubated at 30°C on a shaker for 5 min, after which 150 µL of substrate solution (60 mM Na_2_HPO_4_, 40 mM NaH_2_PO_4_, 1 mg/mL o-nitrophenyl-β-D-galactoside, and 2.7 µL/mL beta-mercaptoethanol) was added to each well. After sufficient yellowing occurred (around 30 min), 175 µL of stop solution (1 M Na_2_CO_3_) was added to each well and their absorbance at 420 nm was measured. Miller units were calculated using the following formula: 1,000 × (OD_420_ / (OD_600_) × (time in min) × (volume of cells in mL). For detection of very low *lacZ* expression (Fig. 5), overnight cultures were normalized to an *A*_600_ value of 2.0, and 5 µL of these suspensions was used in Miller assays as described above, with a reaction time of approximately 90 min prior to adding stop buffer.

### Transcription start site mapping (5′-RACE)

The *A. fabrum* strain, harboring pKJ218, was cultured in LB supplemented with half-strength Sm, Nm, and 1 mM NiCl_2_ to an *A*_600_ of ~0.8. A cell pellet was then made from 1.5 mL of culture, and this was placed at −80°C for 15 min. The frozen pellet was thawed and resuspended in 50 µL of cold TED solution (20 mM Tris [pH 7.5], 10 mM EDTA [pH 8.0], and 40 mM dithiothreitol [DTT]) and 50 µL of 1% SDS. The resuspended pellet was vortexed briefly and immediately placed on a 70°C heat block for 3 min. One milliliter of TRI Reagent (Trizol) was added to the suspension, inverting vigorously. After ensuring the solution was thoroughly mixed, 200 µL of chloroform was added and vortexed briefly, and the suspension was centrifuged for 2 min at 15,000 rpm. After centrifugation, the aqueous layer was separated and placed on ice. Six hundred microliters of isopropanol was then added to the aqueous layer to precipitate the RNA. The RNA suspension was kept on ice for 5 min and then centrifuged. After centrifugation, the supernatant was removed, and the pellet was then resuspended in 40 µL of RNAse-free water and placed in a 70°C heat block. Approximately 1 µg of RNA was reverse transcribed into cDNA using 6 µM gene-specific primer oKJ591, 5× ProtoScript II Buffer, 0.5 mM dNTPs, 10 µM DTT, and 200 U of ProtoScript II Reverse Transcriptase (NEB M0368). The mixture was incubated at 42°C for 1 h. After incubation, the product was purified using a Zymo ZR Plasmid Miniprep-Classic kit (D4016), and the cDNA was eluted in 30 µL of TE (Tris 3 mM and EDTA 0.3 mM). Polyadenylate was added to the 3′ ends of the cDNA molecules using 10× terminal transferase (TdT) buffer, 0.1 mM dATP, 0.25 mM CoCl_2_, and 15 U of TdT (NEB M0315). This was incubated at 37°C for 30 min. The product was placed into a 70°C water bath for 10 min for heat inactivation. The heat-inactivated product was amplified using Taq polymerase (NEB M0267), a gene-specific primer oKJ591, and oKJ595 [this primer consists of a (dT)_18_ at its 3′-end and a G/C rich adapter sequence at its 5′-end]. The amplification protocol included 94°C for 2 min, followed by 10 cycles of 94°C for 15 s, 39°C for 20 s, and 70°C for 45 s; followed by 28 cycles of 94°C for 15 s, 55°C for 20 s, and 70°C for 45 s; and a final extension step at 70°C for 2 min. The product was used in a second round of PCR using a nested primer, oKJ592, along with the (dT)_18_ primer oKJ595. The amplification protocol included 94°C for 2 min, followed by 36 cycles of 94°C for 15 s, 55°C for 20 s, and 70°C for 45 s, and a final extension step at 70°C for 2 min. The product was used in a third round of PCR using a nested primer, oKJ593, along with the (dT)_18_ primer oKJ595. The amplification protocol included 94°C for 2 min, followed by 34 cycles of 94°C for 15 s, 56°C for 20 s, and 70°C for 45 s, and a final extension step at 70°C for 2 min. The product was then cleaned up using a Zymo ZR Plasmid Miniprep-Classic kit (D4016) and submitted for Sanger sequencing using the primer oKJ593.

### Sequence alignments and phylogenetic analysis

An alignment was performed with nucleotide sequences of the region upstream of the *nreAXY* operon acquired from various *Mesorhizobium* strains. Strains and sequences used are listed in Table S3. The sequences were aligned using (ClustalW), and the figure was created in R using ggmsa and WebLogo3 (29, 30). An alignment of NreA/CsoR/RcnR protein sequences (listed in Table S4) was performed using ClustalW, and the figure was created on R using ggmsa (29). A phylogenetic tree reconstruction was built using the Phylogeny.fr analytical server. This online tool used an existing ClustalW-generated multiple sequence alignment of 13 representative amino acid sequences (above) and realigned them using MUSCLE. It then used Gblocks with the highest-stringency settings, which minimizes contiguous strings of non-conserved residues. PhyML (v.3.1/3.0 aLRT) was then used to reconstruct a maximum likelihood-based phylogenetic tree with 100 bootstrap replicates. The WAG substitution model was selected assuming an estimated proportion of invariant sites (of 0.116) and four gamma-distributed rate categories to account for rate heterogeneity across sites. The gamma shape parameter was estimated directly from the data (gamma = 3.991). Reliability for internal branch was assessed using the aLRT test (SH-Like). TreeDyn was used to visualize the tree reconstruction (31–37). An alignment of the NreA/NreA2 protein sequences and the NreX/NreX2 protein sequences (sequences given in the Supplemental Materials) was performed using NCBI BLAST (38).

### Genome-wide search for operator sequence motifs in *Mesorhizobium* C089B

The *Mesorhizobium* C089B genome (NCBI accession number NZ_CP100469.1) was scanned for motifs similar to the *nre* operator. The online motif finder, MEME Suite (v.5.5.8), was used for this search, using the Find Individual Motif Occurrences protocol (39). The *nre* operator sequence (TATCCTCTCGGGGGGGATA) and the degenerate consensus sequence (TATCCYCTCCRGGRGGATA) were each used as input. All matches with a *P* value greater than 1 × 10^−6^ were removed. Hits were then individually checked for their position and surroundings within the genome. Matches that were intragenic were removed. The remaining sequences were tabulated with their genomic position and nearby downstream genes (Table 3).

**TABLE 3:** NreA operator motifs identified in the mesorhizobium C089B genome

Matched sequence	Start	Stop	Strand	Score^*a*^	*P* value^*b*^	*Q* value^*c*^	Region	Upstream	Predicted function
Search with *nreA* operator sequence TATCCTCTCGGGGGGGATA									
TATCCTCTCGGGGGGGATA^*d*^	3,145,188	3,145,206	+	36.6402	2.48E^−12^	1.31E^−5^	Intergenic	*nreA*	Ni-sensing transcriptional regulator
CATCCTCCCCGGGGGGATA	1,073,223	1,073,241	+	15.4756	1.50E^−7^	0.265	Intergenic	*nreA2*	Metal-sensing transcriptional regulator
TCTCCCCCACGAGGGGAGA	4,657,185	4,657,203	+	15.4756	1.50E^−7^	0.265	Intergenic	*glcB*	Malate synthase G
TATCCCCTCCTAGAGGTTT	3,437,282	3,437,300	+	15.2561	2.43E^−7^	0.322	Intergenic	KPGJNAOJ_03459	ATP-dependent nuclease
Search with degenerate consensus sequence (Fig. 4A) TATCCYCTCCRGGRGGATA									
CATCCTCCCCGGGGGGATA	1,073,223	1,073,241	+	23.2744	6.29E^−9^	0.0329	Intergenic	*nreA2*	Metal-sensing transcriptional regulator
TATCCCCCCCGAGAGGATA	3,145,188	3,145,206	+	23.0244	1.12E^−8^	0.0329	Intergenic	*nreA*	Ni-sensing transcriptional regulator
TAACCCCTCGGGGAGGAAA	4,855,066	4,855,084	+	17.7195	1.75E^7^	0.344	Intergenic	*malK*-5	Maltose/maltodextrin import ATP binding
TATCCCCTCCTAGAGGTTT	3,437,282	3,437,300	+	13.3963	6.33E^−7^	0.908	Intergenic	KPGJNAOJ_03459	ATP-dependent nuclease

^*a*^ The score for the match of a position in a sequence to a motif is computed by summing the appropriate entries from each column of the position-dependent scoring matrix that represents the motif.

^*b*^ The *P* value of a motif occurrence is defined as the probability of a random sequence of the same length as the motif matching that position of the sequence with as good or better a score.

^*c*^ The *Q* value of a motif occurrence is defined as the false discovery rate if the occurrence is accepted as significant.

^*d*^ The underlined characters match the associated search sequence.

### NreA structural modeling

NreA structural models were generated using AlphaFold 3 (40). Four copies of NreA were selected to generate the tetrameric structure. To model the apo form of NreA (Fig. S2A), no ligands or ions were selected. To model the metal-bound form, Co(II) was selected as the best of the available choices in the AlphaFold 3 user interface. The DNA-bound version of NreA (Fig. S2C and D) included the operator sequence TATCCTCTCCAGGAGGATA. For each model, pTM scores (which reflect the likelihood of the protein fold being correct, with scores above 0.5 being of high confidence) were as follows: apo-NreA: 0.71, metal-NreA: 0.79, and DNA-NreA: 0.48. The apo form was aligned with an X-ray crystal structure of CsoR from *T. thermophilus* HB8 (PDB 3AAI) using PyMOL (v.3.1.3.1 [Schrödinger, LLC]) with an RMS score of 1.443.

## Data Availability

An annotated GenBank file for reporter plasmid pKJ138 (the starting point for all analyses in this report) may be found under accession number PX233068.

## SUPPLEMENTAL MATERIAL

Supplemental material aem.01403-25-s0001.pdf Figures S1 to S4, Tables S1 to S4, and sequences.

ASM does not own the copyrights to Supplemental Material that may be linked to, or accessed through, an article. The authors have granted ASM a non-exclusive, world-wide license to publish the Supplemental Material files. Please contact the corresponding author directly for reuse.

## References

[1] Macomber L, Hausinger RP. 2011. Mechanisms of nickel toxicity in microorganisms. Metallomics 3:1153–1162. doi:10.1039/c1mt00063b 21799955 PMC4130172

[2] Nies DH. 2003. Efflux-mediated heavy metal resistance in prokaryotes. FEMS Microbiol Rev 27:313–339. doi:10.1016/S0168-6445(03)00048-2 12829273

[3] Chandrangsu P, Rensing C, Helmann JD. 2017. Metal homeostasis and resistance in bacteria. Nat Rev Microbiol 15:338–350. doi:10.1038/nrmicro.2017.15 28344348 PMC5963929

[4] Porter SS, Rice KJ. 2013. Trade-offs, spatial heterogeneity, and the maintenance of microbial diversity. Evolution (N Y) 67:599–608. doi:10.1111/j.1558-5646.2012.01788.x 23356631

[5] Giedroc DP, Arunkumar AI. 2007. Metal sensor proteins: nature’s metalloregulated allosteric switches. Dalton Trans 10:3107–3120. doi:10.1039/b706769k 17637984

[6] Baksh KA, Zamble DB. 2020. Allosteric control of metal-responsive transcriptional regulators in bacteria. J Biol Chem 295:1673–1684. doi:10.1074/jbc.REV119.011444 31857375 PMC7008368

[7] Huang H-T, Bobst CE, Iwig JS, Chivers PT, Kaltashov IA, Maroney MJ. 2018. Co(II) and Ni(II) binding of the Escherichia coli transcriptional repressor RcnR orders its N terminus, alters helix dynamics, and reduces DNA affinity. J Biol Chem 293:324–332. doi:10.1074/jbc.RA117.000398 29150441 PMC5766909

[8] Higgins KA, Giedroc D. 2014. Insights into protein allostery in the CsoR/RcnR family of transcriptional repressors. Chem Lett 43:20–25. doi:10.1246/cl.130965 24695963 PMC3970791

[9] Liu T, Ramesh A, Ma Z, Ward SK, Zhang L, George GN, Talaat AM, Sacchettini JC, Giedroc DP. 2007. CsoR is a novel Mycobacterium tuberculosis copper-sensing transcriptional regulator. Nat Chem Biol 3:60–68. doi:10.1038/nchembio844 17143269

[10] Grossoehme N, Kehl-Fie TE, Ma Z, Adams KW, Cowart DM, Scott RA, Skaar EP, Giedroc DP. 2011. Control of copper resistance and inorganic sulfur metabolism by paralogous regulators in Staphylococcus aureus. J Biol Chem 286:13522–13531. doi:10.1074/jbc.M111.220012 21339296 PMC3075698

[11] Porto TV, Hough MA, Worrall JAR. 2015. Structural insights into conformational switching in the copper metalloregulator CsoR from Streptomyces lividans. Acta Crystallogr D Biol Crystallogr 71:1872–1878. doi:10.1107/S1399004715013012 26327377

[12] Chang F-M, Coyne HJ, Cubillas C, Vinuesa P, Fang X, Ma Z, Ma D, Helmann JD, García-de los Santos A, Wang Y-X, Dann CE III, Giedroc DP. 2014. Cu(I)-mediated allosteric switching in a copper-sensing operon repressor (CsoR). J Biol Chem 289:19204–19217. doi:10.1074/jbc.M114.556704 24831014 PMC4081955

[13] Higgins KA, Hu HQ, Chivers PT, Maroney MJ. 2013. Effects of select histidine to cysteine mutations on transcriptional regulation by Escherichia coli RcnR. Biochemistry 52:84–97. doi:10.1021/bi300886q 23215580 PMC3610428

[14] Ma Z, Jacobsen FE, Giedroc DP. 2009. Coordination chemistry of bacterial metal transport and sensing. Chem Rev 109:4644–4681. doi:10.1021/cr900077w 19788177 PMC2783614

[15] Tan BG, Vijgenboom E, Worrall JAR. 2014. Conformational and thermodynamic hallmarks of DNA operator site specificity in the copper sensitive operon repressor from Streptomyces lividans. Nucleic Acids Res 42:1326–1340. doi:10.1093/nar/gkt902 24121681 PMC3902906

[16] Iwig JS, Leitch S, Herbst RW, Maroney MJ, Chivers PT. 2008. Ni(II) and Co(II) sensing by Escherichia coli RcnR. J Am Chem Soc 130:7592–7606. doi:10.1021/ja710067d 18505253 PMC2435081

[17] Brady KU, Kruckeberg AR, Bradshaw Jr. HD. 2005. Evolutionary ecology of plant adaptation to serpentine soils. Annu Rev Ecol Evol Syst 36:243–266. doi:10.1146/annurev.ecolsys.35.021103.105730

[18] Kehlet-Delgado H, Montoya AP, Jensen KT, Wendlandt CE, Dexheimer C, Roberts M, Torres Martínez L, Friesen ML, Griffitts JS, Porter SS. 2024. The evolutionary genomics of adaptation to stress in wild rhizobium bacteria. Proc Natl Acad Sci USA 121:e2311127121. doi:10.1073/pnas.2311127121 38507447 PMC10990125

[19] Iwig JS, Chivers PT. 2009. DNA recognition and wrapping by Escherichia coli RcnR. J Mol Biol 393:514–526. doi:10.1016/j.jmb.2009.08.038 19703465

[20] Porter SS, Chang PL, Conow CA, Dunham JP, Friesen ML. 2017. Association mapping reveals novel serpentine adaptation gene clusters in a population of symbiotic Mesorhizobium. ISME J 11:248–262. doi:10.1038/ismej.2016.88 27420027 PMC5315480

[21] Dwarakanath S, Chaplin AK, Hough MA, Rigali S, Vijgenboom E, Worrall JAR. 2012. Response to copper stress in Streptomyces lividans extends beyond genes under direct control of a copper-sensitive operon repressor protein (CsoR). J Biol Chem 287:17833–17847. doi:10.1074/jbc.M112.352740 22451651 PMC3366776

[22] Ma Z, Cowart DM, Scott RA, Giedroc DP. 2009. Molecular insights into the metal selectivity of the copper(I)-sensing repressor CsoR from Bacillus subtilis. Biochemistry 48:3325–3334. doi:10.1021/bi900115w 19249860 PMC2728441

[23] Grabowska AD, Andreu N, Cortes T. 2021. Translation of a leaderless reporter is robust during exponential growth and well sustained during stress conditions in Mycobacterium tuberculosis. Front Microbiol 12:746320. doi:10.3389/fmicb.2021.746320 34603273 PMC8485053

[24] Shell SS, Wang J, Lapierre P, Mir M, Chase MR, Pyle MM, Gawande R, Ahmad R, Sarracino DA, Ioerger TR, Fortune SM, Derbyshire KM, Wade JT, Gray TA. 2015. Leaderless transcripts and small proteins are common features of the mycobacterial translational landscape. PLoS Genet 11:e1005641. doi:10.1371/journal.pgen.1005641 26536359 PMC4633059

[25] Hynes MF, Simon R, Pühler A. 1985. The development of plasmid-free strains of Agrobacterium tumefaciens by using incompatibility with a Rhizobium meliloti plasmid to eliminate pAtC58. Plasmid 13:99–105. doi:10.1016/0147-619x(85)90062-9 4001194

[26] Hanahan D. 1983. Studies on transformation of Escherichia coli with plasmids. J Mol Biol 166:557–580. doi:10.1016/s0022-2836(83)80284-8 6345791

[27] Griffitts JS, Carlyon RE, Erickson JH, Moulton JL, Barnett MJ, Toman CJ, Long SR. 2008. A Sinorhizobium meliloti osmosensory two-component system required for cyclic glucan export and symbiosis. Mol Microbiol 69:479–490. doi:10.1111/j.1365-2958.2008.06304.x 18630344

[28] Zhang X, Bremer H. 1995. Control of the Escherichia coli rrnB P1 promoter strength by ppGpp. J Biol Chem 270:11181–11189. doi:10.1074/jbc.270.19.11181 7538113

[29] Zhou L, Feng T, Xu S, Gao F, Lam TT, Wang Q, Wu T, Huang H, Zhan L, Li L, Guan Y, Dai Z, Yu G. 2022. ggmsa: a visual exploration tool for multiple sequence alignment and associated data. Brief Bioinformatics 23. doi:10.1093/bib/bbac222 35671504

[30] Crooks GE, Hon G, Chandonia JM, Brenner SE. 2004. WebLogo: a sequence logo generator. Genome Res 14:1188–1190. doi:10.1101/gr.849004 15173120 PMC419797

[31] Dereeper A, Guignon V, Blanc G, Audic S, Buffet S, Chevenet F, Dufayard J-F, Guindon S, Lefort V, Lescot M, Claverie J-M, Gascuel O. 2008. Phylogeny.fr: robust phylogenetic analysis for the non-specialist. Nucleic Acids Res 36:W465–W469. doi:10.1093/nar/gkn180 18424797 PMC2447785

[32] Chevenet F, Brun C, Bañuls A-L, Jacq B, Christen R. 2006. TreeDyn: towards dynamic graphics and annotations for analyses of trees. BMC Bioinformatics 7:439. doi:10.1186/1471-2105-7-439 17032440 PMC1615880

[33] Anisimova M, Gascuel O. 2006. Approximate likelihood-ratio test for branches: a fast, accurate, and powerful alternative. Syst Biol 55:539–552. doi:10.1080/10635150600755453 16785212

[34] Castresana J. 2000. Selection of conserved blocks from multiple alignments for their use in phylogenetic analysis. Mol Biol Evol 17:540–552. doi:10.1093/oxfordjournals.molbev.a026334 10742046

[35] Dereeper A, Audic S, Claverie J-M, Blanc G. 2010. BLAST-EXPLORER helps you building datasets for phylogenetic analysis. BMC Evol Biol 10:8. doi:10.1186/1471-2148-10-8 20067610 PMC2821324

[36] Edgar RC. 2004. MUSCLE: multiple sequence alignment with high accuracy and high throughput. Nucleic Acids Res 32:1792–1797. doi:10.1093/nar/gkh340 15034147 PMC390337

[37] Guindon S, Gascuel O. 2003. A simple, fast, and accurate algorithm to estimate large phylogenies by maximum likelihood. Syst Biol 52:696–704. doi:10.1080/10635150390235520 14530136

[38] NCBI Resource Coordinators. 2016. Database resources of the national center for biotechnology information. Nucleic Acids Res 44:D7–D19. doi:10.1093/nar/gkv1290 26615191 PMC4702911

[39] Bailey TL, Johnson J, Grant CE, Noble WS. 2015. The MEME suite. Nucleic Acids Res 43:W39–W49. doi:10.1093/nar/gkv416 25953851 PMC4489269

[40] Abramson J, Adler J, Dunger J, Evans R, Green T, Pritzel A, Ronneberger O, Willmore L, Ballard AJ, Bambrick J, et al.. 2024. Accurate structure prediction of biomolecular interactions with AlphaFold 3. Nature 630:493–500. doi:10.1038/s41586-024-07487-w 38718835 PMC11168924

