# Supplementary material for: Regulation of a nickel tolerance operon conserved in Mesorhizobium strains from serpentine soils
Source: Appl Environ Microbiol. 2025 Nov 4;91(12):e01403-25. doi: 10.1128/aem.01403-25 (PMC12724321; doi:10.1128/aem.01403-25)
Supplement: Supplemental material — Figures S1 to S4, Tables S1 to S4, and sequences. [file aem.01403-25-s0001.pdf]

Supplemental Materials for "Regulation of a nickel tolerance operon conserved in *Mesorhizobium* strains from serpentine soils"

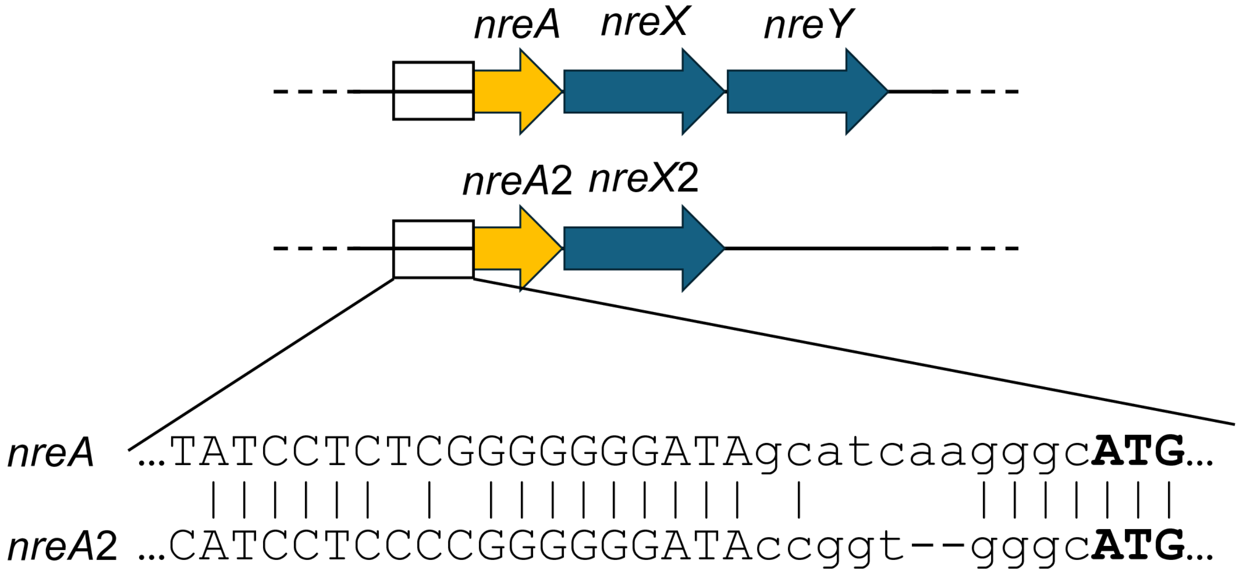

Figure S1. Comparison of the *nreAXY* operon and *nreA2X2* operon in *Mesorhizobium* C089B, with emphasis on the upstream operator sequences in each. Start codons are in bold.

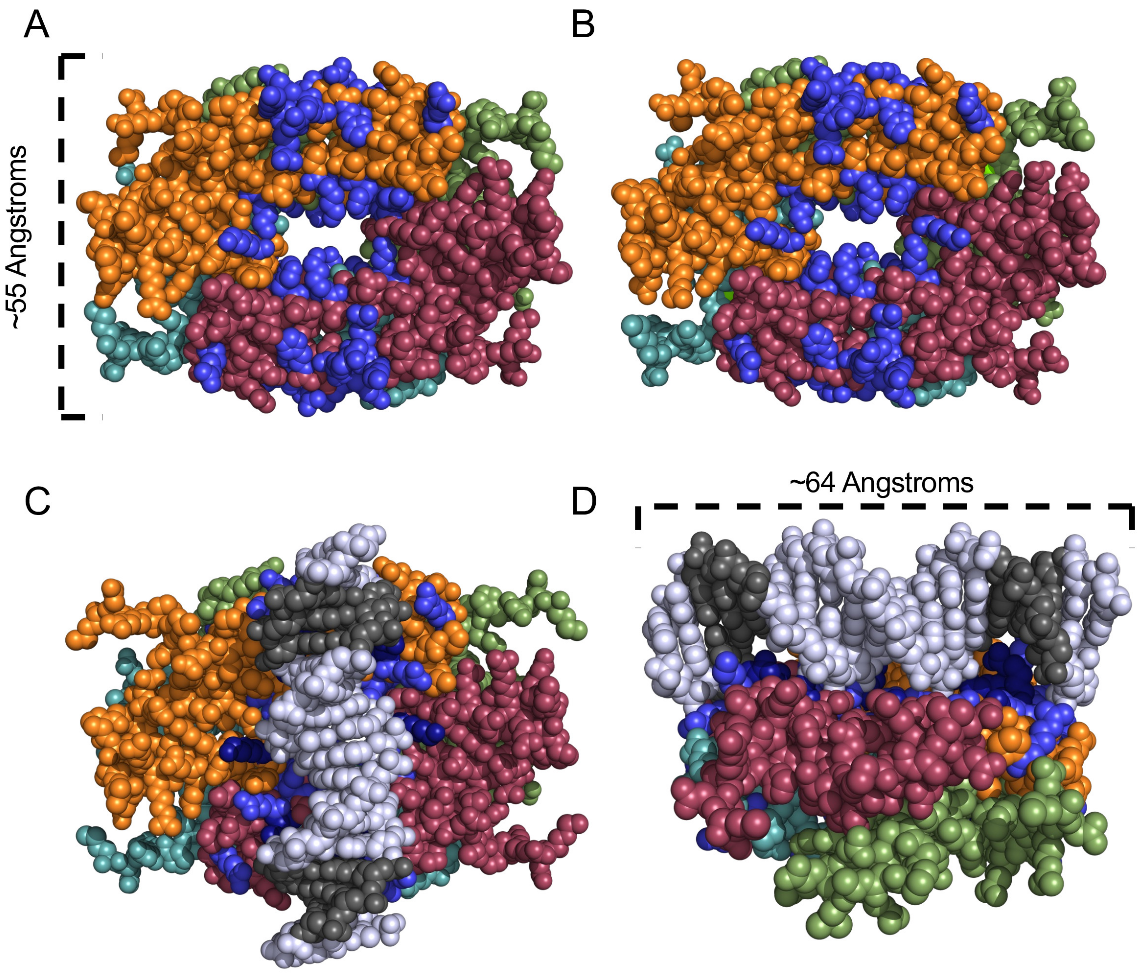

Figure S2. Predicted structures of apo, metal-bound, and DNA-bound NreA. NreA homotetramer subunits are colored (orange, red, green, and cyan). (**A**) Apo form of NreA tetramer, blue residues represent positively charged residues located on a proposed DNA-binding surface. The approximate length of the proposed DNA-binding surface is given. (**B**) Predicted metal-bound form of the NreA tetramer. (**C,D**) Two views of the predicted DNA-bound structure of NreA. The optimized operator sequence TATCCTCTCCAGGAGGATA was used. DNA bases colored in dark gray represent the most important residues (TCC/GGA) based on the experiment in Figure 4. The approximate length of the operator DNA is shown.

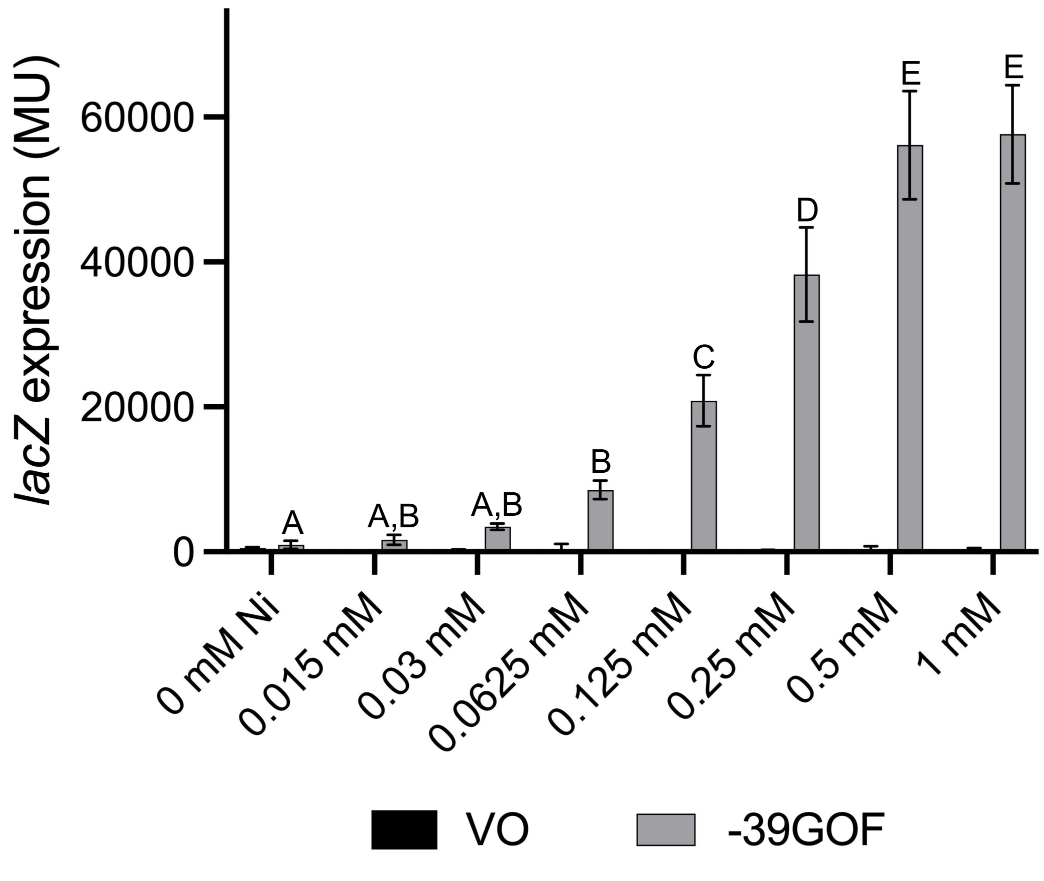

Figure S3. Reporter gene expression from the -39GOF promoter as a function of $NiCl_2$ concentration. *A. fabrum* was used for this experiment; VO, vector-only control strain. β-galactosidase activity was measured in MU. Error bars represent standard deviation from the mean (n=5). A two-way ANOVA was performed, followed by Tukey's multiple comparisons test. If two data points have different letters, they are significantly different.

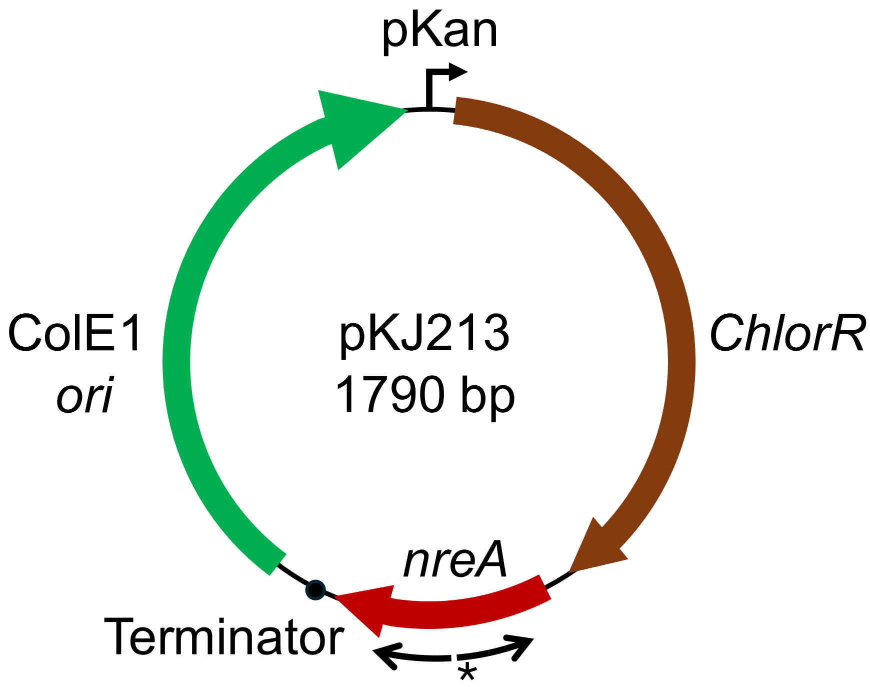

Figure S4. Map of pKJ213, used to introduce alanine substitutions into *nreA*. Black arrows underneath *nreA* represent primers used to introduce alanine substitutions into *nreA* via inverse-PCR prior to insertion into reporter plasmid pKJ227.

Table S1. Strains used in this study

Strain	Genotype	Purpose/Description[d]
B001	Cm^R	DH5α containing the pRK600 plasmid from strain MT616.
C089B		Wild-type *Mesorhizobium* strain C089B.
D224	Sm^R	*Agrobacterium fabrum* strain UBAPF2. Has been selected for Sm^R.
KJ003	*gyrA96*	*E. coli* (DH5α). Resistant to Nalidixic acid.
KJ212	Sm^R, Nm^R	D224 (*A. fabrum*) with pKJ138 (-*nreA)*.
KJ213	Sm^R, Nm^R	D224 (*A. fabrum*) with pKJ140 (+*nreA)*.
KJ269[a]	Sm^R, Nm^R	D224 (*A. fabrum*) with pKJ217.
KJ270[a]	Sm^R, Nm^R	D224 (A. fabrum) with pKJ218.
KJ271[a]	Sm^R, Nm^R	D224 (*A. fabrum*) with pKJ219.
KJ272[a]	Sm^R, Nm^R	D224 (*A. fabrum*) with pKJ220.
KJ273[a]	Sm^R, Nm^R	D224 (*A. fabrum*) with pKJ221.
KJ274[a]	Sm^R, Nm^R	D224 (*A. fabrum*) with pKJ222.
KJ277[b]	Sm^R, Nm^R	D224 (*A. fabrum*) with pKJ225.
KJ278[b]	Sm^R, Nm^R	D224 (*A. fabrum*) with pKJ226.
KJ279[b]	Sm^R, Nm^R	D224 (*A. fabrum*) with pKJ227.
KJ280[b]	Sm^R, Nm^R	D224 (*A. fabrum*) with pKJ228.
KJ283[b]	Sm^R, Nm^R	D224 (*A. fabrum*) with pKJ231.
KJ284[b]	Sm^R, Nm^R	D224 (*A. fabrum*) with pKJ232.
KJ285[b]	Sm^R, Nm^R	D224 (*A. fabrum*) with pKJ233.
KJ286[b]	Sm^R, Nm^R	D224 (*A. fabrum*) with pKJ234.
KJ287[b]	Sm^R, Nm^R	D224 (*A. fabrum*) with pKJ235.
KJ289[b]	Sm^R, Nm^R	D224 (*A. fabrum*) with pKJ238.
KJ291[b]	Sm^R, Nm^R	D224 (*A. fabrum*) with pKJ240.
KJ293[b]	Sm^R, Nm^R	D224 (*A. fabrum*) with pKJ242.
KJ295[b]	Sm^R, Nm^R	D224 (*A. fabrum*) with pKJ244.
KJ297[b]	Sm^R, Nm^R	D224 (*A. fabrum*) with pKJ246.
KJ305[c]	Sm^R, Nm^R	D224 (*A. fabrum*) with pKJ257 (wt *nreA)*.
KJ306[c]	Sm^R, Nm^R	D224 (*A. fabrum*) with pKJ258 (C38A).
KJ307[c]	Sm^R, Nm^R	D224 (*A. fabrum*) with pKJ259 (H63A).
KJ308[c]	Sm^R, Nm^R	D224 (*A. fabrum*) with pKJ260 (R17A).
KJ309[c]	Sm^R, Nm^R	D224 (*A. fabrum*) with pKJ261 (H66A).
KJ312[c]	Sm^R, Nm^R	D224 (*A. fabrum*) with pKJ264 (Q44A).
KJ313[c]	Sm^R, Nm^R	D224 (*A. fabrum*) with pKJ265 (C67A).
KJ315[c]	Sm^R, Nm^R	D224 (*A. fabrum*) with pKJ267 (R20A).
KJ316[c]	Sm^R, Nm^R	D224 (*A. fabrum*) with pKJ268 (K91A).

[a]Strains used in promoter truncation analysis
[b]Strains used in promoter/operator mutation analysis
[c]Strains used in NreA mutation analysis
[d]Refer to Table 1 for the descriptions of the variant promoters/operators used

Table S2. Primers used in this study

Primer	Sequence (5'-3')	Purpose/Description
oKJ498	GATCCGGCTTGGAATATCCTCTCGGGGGGGATAGC ATCAAGGGCAT	Top oligo for -39 truncation of *nre* promoter region from C089B. Anneal with oKJ499.
oKJ499	CTAGATGCCCTTGATGCTATCCCCCCCGAGAGGAT ATTCCAAGCCG	Bottom oligo for -39 truncation of *nre* promoter region from C089B. Anneal with oKJ498.
oKJ502	GATCCGGCAACGAATATCCTCTCGGGGGGGATAGC ATCAAGGGCAT	Top oligo for -36TTG>AAC, in nre operator sequence. Anneal with oKJ503.
oKJ503	CTAGATGCCCTTGATGCTATCCCCCCCGAGAGGAT ATTCGTTGCCG	Bottom oligo for -36TTG>AAC, in nre operator sequence. Anneal with oKJ502.
oKJ504	GATCCGGCTTGAAATATCCTCTCGGGGGGGATATA ATCAAGGGCAT	Top oligo for -33G>A; -11GC>TA, in nre operator sequence. Anneal with oKJ505.
oKJ505	CTAGATGCCCTTGATTATATCCCCCCCGAGAGGAT ATTTCAAGCCG	Bottom oligo for -33G>A; -11GC>TA, in nre operator sequence. Anneal with oKJ504.
oKJ527	GCGGGATCCAATTCGGCATG	Forward primer to amplify -120 truncation of *nre* promoter region from C089B. Use with oKJ564.
oKJ554	GATCCGGCTTGAAATAAAATCTCGGGGGGGATATA ATCAAGGGCAT	Top oligo for -28TCC>AAA in *nre* operator sequence. Anneal with oKJ555.
oKJ555	CTAGATGCCCTTGATTATATCCCCCCCGAGATTTT ATTTCAAGCCG	Bottom oligo for -28TCC>AAA in *nre* operator sequence. Anneal with oKJ554.
oKJ556	GATCCGGCTTGAAATATCCAAACGGGGGGGATATA ATCAAGGGCAT	Top oligo for -25TCT>AAA in *nre* operator sequence. Anneal with oKJ557.
oKJ557	CTAGATGCCCTTGATTATATCCCCCCCGTTTGGAT ATTTCAAGCCG	Bottom oligo for -25TCT>AAA in *nre* operator sequence. Anneal with oKJ556.
oKJ558	GATCCGGCTTGAAATATCCTCTAAAGGGGGATATA ATCAAGGGCAT	Top oligo for -22CGG>AAA in *nre* operator sequence. Anneal with oKJ559.
oKJ559	CTAGATGCCCTTGATTATATCCCCCTTTAGAGGAT ATTTCAAGCCG	Bottom oligo for -22CGG>AAA in *nre* operator sequence. Anneal with oKJ558.
oKJ560	GATCCGGCTTGAAATATCCTCTCGGAAAGGATATA ATCAAGGGCAT	Top oligo for -19GGG>AAA in *nre* operator sequence. Anneal with oKJ561.
oKJ561	CTAGATGCCCTTGATTATATCCTTTCCGAGAGGAT ATTTCAAGCCG	Bottom oligo for -19GGG>AAA in *nre* operator sequence. Anneal with oKJ560.
oKJ562	GATCCGGCTTGAAATATCCTCTCGGGGGAAATATA ATCAAGGGCAT	Top oligo for -16GG>AA in *nre* operator sequence. Anneal with oKJ563.
oKJ563	CTAGATGCCCTTGATTATATTTCCCCCGAGAGGAT ATTTCAAGCCG	Bottom oligo for -16GG>AA in *nre* operator sequence. Anneal with oKJ562.
oKJ564	GCGTCTAGATGCCCTTGATGCTATCCC	Reverse primer to amplify -120 truncation of *nre* promoter region from C089B. Anneal with oKJ527.
oKJ565	GATCCGGCTTGAAATATCCTCTCGGGGAGGATATA ATCAAGGGCAT	Top oligo for -17G>A in *nre* operator sequence. Anneal with oKJ566.

oKJ566	CTAGATGCCCTTGATTATATCCTCCCCGAGAGGAT ATTTCAAGCCG	Bottom oligo for -17G>A in *nre* operator sequence. Anneal with oKJ565.
oKJ567	GATCCGGCTTGAAATATCCTCTCCAGGGGGATATA ATCAAGGGCAT	Top oligo for -21GG>CA in *nre* operator sequence. Anneal with oKJ568.
oKJ568	CTAGATGCCCTTGATTATATCCCCCTGGAGAGGAT ATTTCAAGCCG	Bottom oligo for -21GG>CA in *nre* operator sequence. Anneal with oKJ567.
oKJ569	GATCCGGCTTGAAATATCCTCTCCAGGAGGATATA ATCAAGGGCAT	Top oligo for -21GG>CA;-17G>A in *nre* operator sequence. Anneal with oKJ570.
oKJ570	CTAGATGCCCTTGATTATATCCTCCTGGAGAGGAT ATTTCAAGCCG	Bottom oligo for -21GG>CA;-17G>A in *nre* operator sequence. Anneal with oKJ569.
oKJ571	GATCCGGCTTGAAATATCCCCTCGGGGAGGATATA ATCAAGGGCAT	Top oligo for -25T>C;-17G>A in *nre* operator sequence. Anneal with oKJ572.
oKJ572	CTAGATGCCCTTGATTATATCCTCCCCGAGGGGAT ATTTCAAGCCG	Bottom oligo for -25T>C;-17G>A in *nre* operator sequence. Anneal with oKJ571.
oKJ573	GATCCGGCTTGAAATATCCCCTCCAGGGGGATATA ATCAAGGGCAT	Top oligo for -25T>C;-21GG>CA in *nre* operator sequence. Anneal with oKJ574.
oKJ574	CTAGATGCCCTTGATTATATCCCCCTGGAGGGGAT ATTTCAAGCCG	Bottom oligo for -25T>C;-21GG>CA in *nre* operator sequence. Anneal with oKJ573.
oKJ575	GATCCTATCCTCTCGGGGGGGATAGCATCAAGGGC AT	Top oligo for -30 truncation of *nre* promoter region from C089B. Anneal with oKJ576.
oKJ576	CTAGATGCCCTTGATGCTATCCCCCCCGAGAGGAT AG	Bottom oligo for -30 truncation of *nre* promoter region from C089B. Anneal with oKJ575.
oKJ581	CGGTCGCCCGGCCTC	Forward primer to introduce C38A mutation in NreA. Use with oKJ582.
oKJ582	GCGCTCGACATCGCCCAGC	Reverse primer to introduce C38A mutation in NreA. Use with oKJ581.
oKJ583	GTCCTGGATCAGCGTCTTC	Forward primer to introduce H63A mutation in NreA. Use with oKJ584.
oKJ584	GCGCTCAACCACTGCCTTGAAG	Reverse primer to introduce H63A mutation in NreA. Use with oKJ583.
oKJ585	CTTGATGATGGCAGGATGG	Forward primer to introduce R17A mutation in NreA. Use with oKJ586.
oKJ586	GCGCTGAAGCGCGCCGACG	Reverse primer to introduce R17A mutation in NreA. Use with oKJ585.
oKJ591	cgagtaacaacccgtcg	lacZ specific gene primer for 5' RACE.
oKJ592	ggattctccgtgggaac	Nested 1 lacZ specific gene primer for 5' RACE.
oKJ593	cgcAAGCTTgcggattgaccgtaatgg	Nested 2 lacZ specific gene primer for 5' RACE. Brings in HindIII.
oKJ595	CGCGGATCCTCTAGATTTTTTTTTTTTTTTTTT	Anchor primer for 5' and 3' RACE. Contains additional GC-rich sequence (BamHI/XbaI sites) at its 5' end.

oKJ596	GCGTGCCTTGAAGACGTCGTC	Forward primer to introduce H66A mutation in NreA. Use with oKJ597.
oKJ597	GTTGAGGTGGTCCTGGATC	Reverse primer to introduce H66A mutation in NreA. Use with oKJ597.
oKJ604	GCGcttcatgccgtggagaag	Forward primer to introduce Q44A mutation in nreA. Use with oKJ605. 130CAA>GCG.
oKJ605	ctgggcgatgtcgagG	Reverse primer to introduce Q44A mutation in nreA. Use with oKJ604.
oKJ606	gcgCTTGAAGACGTCGTCGGAC	Forward primer to introduce C67A mutation in nreA. Use with oKJ607. 199TGC>GCG.
oKJ607	GTGgttgagGTGgtcctg	Reverse primer to introduce C67A mutation in nreA. Use with oKJ606.
oKJ608	GAATGCctcgacatcgccc	Forward primer to introduce P37E mutation in nreA. Use with oKJ609. 109CCG>GAA.
oKJ609	tcgcccggcctcgatc	Reverse primer to introduce P37E mutation in nreA. Use with oKJ608.
oKJ610	GCGgccgacggccatctgc	Forward primer to introduce R20A mutation in nreA. Use with oKJ611. 58CGC>GCG.
oKJ611	cttcagccgcttgatgatg	Reverse primer to introduce R20A mutation in nreA. Use with oKJ610.
oKJ612	GCGtacctgtgaGGTaAgcttag	Forward primer to introduce K91A mutation in nreA. Use with oKJ613. 271AA>GC.
oKJ613	ggtgatatccttgaactcgtc	Reverse primer to introduce K91A mutation in nreA. Use with oKJ612.
oKJ614	GCGggtACCaggaggtatacatATG	Forward primer to amplify nreA mutants for insertion into pKJ228. Use with oKJ615.
oKJ615	GCTGAggtACCtcacagg	Reverse primer to amplify nreA mutants for insertion into pKJ228. Use with oKJ614.

Table S3. ***Mesorhizobium*** **alignment naming key**

Name	NreA Accession	Genome Accession	Species	Genomic Region (Start)
Meso 1	WP_184874804.1	NZ_JACHEF010000004.1	*M. sangaii*	485,058
Meso 2	WP_127385817.1	NZ_SMYZ01000087.1	*M. norvegicum*	33,165
Meso 3	WP_023679360.1	NZ_AYVL01000026.1 NZ_AYVL01000000	*M. sp.*	13,979
Meso 4	WP_023680176.1	NZ_AYVL01000040 NZ_AYVL01000000	*M. sp.*	19,816
Meso 5	WP_023722121.1	NZ_AYVY01000034.1 NZ_AYVY01000000	*M. sp.*	26,134
Meso 6	WP_023758556.1	NZ_AYWO01000005.1 NZ_AYWO01000000	*M. sp.*	311,826
Meso 7	WP_023785294.1	NZ_AYWS01000037.1 NZ_AYWS01000000	*M. sp.*	4,072
Meso 8	WP_023802373.1	NZ_AYWU01000025.1 NZ_AYWU01000000	*M. sp.*	31,825
Meso 9	WP_023809580.1	NZ_AYWX01000002.1 NZ_AYWX01000000	*M. sp.*	262,506
Meso 10	WP_023813372.1	NZ_AYWX01000014.1 NZ_AYWX01000000	*M. sp.*	104,752
Meso 11	WP_095081632.1	NZ_NNRI01000004.1 NZ_NNRI01000000	*M. sophorae*	78,561
Meso 12	WP_120017144.1	NZ_QZWZ01000024.1 NZ_QZWZ01000000	*M. waimense*	83,400
Meso 13	WP_123150398.1	NZ_FUIG01000044.1 NZ_FUIG01000000	*M. delmotii*	100,349
Meso 14	WP_127221317.1	NZ_RZSC01000282.1 NZ_RZSC01000000	*M. sp.*	838
Meso 15	WP_127292000.1	NZ_RZRT01000006.1 NZ_RZRT01000000	*M. sp.*	42,794
Meso 16	WP_214473723.1	NZ_JAFFIW010000023.1 NZ_JAFFIW010000000	*M. sp.*	51,304
Meso 17	WP_128304862.1	NZ_RZRV01000025.1 NZ_RZRV01000000	*M. sp.*	10,949
Meso 18	WP_129411591.1	NZ_CP029562.1	*M. sp.*	1,524,238
Meso 19	WP_183432071.1	NZ_JACHWN010000006.1 NZ_JACHWN010000000	*M. sp.*	12,725
Meso 20	OJU50141.1	MKRZ01000089.1 MKRZ01000000	*M. sp.*	65,039
Meso 21	WP_256752877.1	NZ_JALJRL010000013.1 NZ_JALJRL010000000	*M. sp.*	122,141

Table S4. NreA/CsoR/RcnR sequences used for phylogenetic comparison

Name	NCBI Accession	Species	Strain	Protein sequence
CsoR	4M1P_A (PDB)	*Geobacillus thermodenitrificans*	NG80-2	AHPSQEEHVLHGTMIPRTKEEIENIMKRLKRIEGQVRGVQKMVEDNRYCIDILVQISAIQAALRQVGMQLLERHANHCVAKAIREGSGEQSLRELMDVIKQFAK
CsoR	WP_003404935.1	*Mycobacterium tuberculosis*	H37RV	MSKELTAKKRAALNRLKTVRGHLDGIVRMLESDAYCVDVMKQISAVQSSLERANRVMLHNHLETCFSTAVLDGHGQAAIEELIDAVKFTPALTGPHARLGGAAVGESATEEPMPDASNM
CsoR	WP_003228404.1	*Bacillus subtilis*	CU1065_W168	MEKHNEHKTLNHKSSKEKDQITNRLKRIEGQVRGIQNMVENDRYCVDILVQISAVQAAMKNVALHLLEDHAHHCVADAIKSGDGEQAISELLDVFKKFTKS
CsoR	WP_011173741.1	*Thermus thermophilus*	HB8_TTHA1719	MPHSHLHLDPKVREEARRRLLSAKGHLEGILRMLEDEKVYCVDVLKQLKAVEGALDRVGEMVLRAHLKDHVATAHERGDVEEIVEELMEALKYR
CsoR	WP_008633933.1	*Thermus thermophilus*	HB8_TTHA1953	MTRTTDLGQETVDNILKRLRRIEGQVRGLQKMVAEGRPCDEVLTQMTATKKAMEAAATLILHEFLNVCAAEVSEGKVNPKKPEEIATMLKKFI
NreA	AAA72440.1	*Alcaligenes xylosoxidans*	31A	MGVHTSHASIIKRLKRAEGHLRSIVVMMEEGRPCLAIAQQLQAVESAVTQAKKALVHDHIDHCLEEAVRDGTRPSDETLREFKSITKYL
NreA	EIM72205.1	*Nitratireductor aquibiodomus*	RA22	MNTQPHLHETHPQIVKRLKRADGHLRGVIEMIEAGRPCLDIAQQLHAVEKAIAQAKKTLIQDHLDHCLEEVVGPMTRERRQSIDEFREIAKYL
NreA	MDH6264150.1	*Bradyrhizobium sp.*	BR13661	MRDHSHLAIARRLKRANGHLESIIEMVENNRPCAEIAQQLQAVESAIESAKKTMIHDHISHSLERSFKTHGQKGQAALRDFKLISKYL
NreA	WP_023669876	*Mesorhizobium sp.*	C089B	MNERPHVHETHPAIIKRLKRADGHLRGIVEMIEAGRPCLDIAQQLHAVEKAIAQAKKTLIQDHLNHCLEDVVGPLALEQRRSIDEFKDITKYL
NreA	RUW97629.1	*Mesorhizobium sp.*	M8	MSEKPHIHETHPDIVKRLKRANGHLKGVIEMIEAGRPCLDIAQQLHAVEKAISQAKKTLIQDHLNHCLEDVVGPLAREQRRSIDEFKDIAKYL
RcnR	WP_012134972.1	*Citrobacter koseri*	BAA_895	MSHTIRDKQKLKARASKIQGQVVALKKMLDEPHECAAVLQQIAAIRGAVNGLMREVIKGHLTEHIVHQSDEVKREDDLEVILKVLDSYIK
RcnR	WP_000019953.1	*Salmonella paratyphi*	BAA_1250	MSHTIRDKQKLKARTSKIQGQVIALKKMLDEPHECAAVLQQIAAIRGAVNGLMREVIKGHLTEHIVHQSDEARREEDLDVILKVLDSYIK

RcnR	Q0T334.1	*Shigella flexneri*	5B	MSHTIRDKQKLKARASKIQGQVVALKKMLDEP HECAAVLQQIAAIRGAVNGLMREVIKGHLTEH IVHQGDELKREEDLDVVLKVLDSYIK
RcnR	WP_181193565.1	*Eschershia coli*	BW25113	MSHTIRDKQKLKARASKIQGQVVALKKMIDEP HECAAVLQQIAAIRGAVNGLMREVIKGHLTEH IVHQGDELKREEDLDVVLKVLDSYIK

Protein sequences in the two homologous *nreA* operons in *Mesorhizobium* strain C089B

NCBI accession NZ_CP100469.1

NreA (93 AA)

```
MNERPHVHETHPAIIKRLKRADGHLRGIVEMIEAGRPCLDIAQQLHAVEKAIAQAKKTLIQDHLNHCLED
VVGPLALEQRRSIDEFKDITKYL*
```

NreA2 (86 AA)

```
MSEGIHSSHPAIVKRLKRAQGHLASVLGMFEAQRSCLDLAQQLHAVESAISSAKRELIHDHIEHCLIDTE
AGGNSLAELKQLAKYL*
```

NreX (446 AA)

```
MMDKVRDWFGFGPMTQGMGGHGHDHGEGGHGHTHGVIDPTIATTTRGIWAIKWSFVVLAITAALQLVVVF
LSGSVALLADTIHNVGDAVTAIPLWVAFMLARRKPSKTFTYGLGRVEDLAGILIVLIILFSAIVAGYEAI
DRLINPQPIAFLGWVAIAGIIGFLGNEAVAVFRIRVGREINSAALIADGYHARTDGFTSLAVVLGAIGVW
LGFPLADPIIGLLITVAIFAIVWQSSKAVLTRMLDGVEPGIVDEIHHAAEHVSGIERVQSVQARWIGHRL
HADVAISVADAATAKDVMGVTEALKEELFAHLPALAEANVRLESSAGSAATGTEPSHSHGHHAPEPFKVD
CDLAAGTLEIVDTPAGERMRLTIDRHADDLMATVIIDRPGGPETLRLEPIADNHHRLESTVAPAEPHEFQ
ARLVLAAKGREQVLPFKMVEPEGHHH*
```

NreX2 (343 AA)

```
MTSIGARVLDWFGFGSHDHDAHAHDDGSHGHTHGVIDATIATTDRGIWAIKWSFVILAMTAALQMAVVLV
SRSVALLADTIHNIGDATTAIPLWIAFMLARRKPSRTFSYGLGRVEDLAGIIIVLIILFSAIVAGYQAIE
RLVSPQAVTHLGWLTAAGIVGFLGNEAVAVFRIRIGREINSAALVADGYHARTDGLTSLAVVIGAMGVWL
GFPLADPIIGLLITVAIFGIVWQSARSVLTRMLDGVEPGVMAEIQHAAAHVQGARVVDAKARWIGHKLHA
DIAIAADGTLPLSEANKITAALENELFEHMPALAAANIRFSTDQGEHEHPHSHDQGEHHGHQH*
```

Annotated sequences for plasmids used in this study

pKJ138 (8199 bp)
Vector used for *lacZ* reporter gene studies; *nreA*$^-$
BamHI: 28-33, XbaI: 37-42 (for promoter/operator cloning)
RBS: 37-54 bp
lacZ: 55-3129 bp
T7hyb1 Terminator: 3162-3203 bp
Km/Nm resistance (*kanR*): 3371-4165 bp
KpnI: 4169-4174 (nreA inserted in pKJ140 and other variants)
p15A *ori*: 4445-4990 bp
RK2 *oriT*: 5219-5328 bp (for conjugation)
pVS1 *ori/sta*: 5601-7991 bp (for *Agrobacterium* replication)

```
gagctcaccgaattcccactcgagccaGGATCCaccTCTAGAAGGAGGTTACATATGACCATGATTACGG
ATTCACTGGCCGTCGTTTTACAACGTCGTGACTGGGAAAACCCTGGCGTTACCCAACTTAATCGCCTTGC
AGCACATCCCCCTTTCGCCAGCTGGCGTAATAGCGAAGAGGCCCGCACCGATCGCCCTTCCCAACAGTTG
CGCAGCCTGAATGGCGAATGGCGCTTTGCCTGGTTTCCGGCACCAGAAGCGGTGCCGGAAAGCTGGCTGG
AGTGCGATCTTCCTGAGGCCGATACTGTCGTCGTCCCCTCAAACTGGCAGATGCACGGTTACGATGCGCC
CATCTACACCAACGTGACCTATCCCATTACGGTCAATCCGCCGTTTGTTCCCACGGAGAATCCGACGGGT
TGTTACTCGCTCACATTTAATGTTGATGAAAGCTGGCTACAGGAAGGCCAGACGCGAATTATTTTTGATG
GCGTTAACTCGGCGTTTCATCTGTGGTGCAACGGGCGCTGGGTCGGTTACGGCCAGGACAGTCGTTTGCC
GTCTGAATTTGACCTGAGCGCATTTTTACGCGCCGGAGAAAACCGCCTCGCGGTGATGGTGCTGCGCTGG
AGTGACGGCAGTTATCTGGAAGATCAGGATATGTGGCGGATGAGCGGCATTTTCCGTGACGTCTCGTTGC
TGCATAAACCGACTACACAAATCAGCGATTTCCATGTTGCCACTCGCTTTAATGATGATTTCAGCCGCGC
TGTACTGGAGGCTGAAGTTCAGATGTGCGGCGAGTTGCGTGACTACCTACGGGTAACAGTTTCTTTATGG
CAGGGTGAAACGCAGGTCGCCAGCGGCACCGCGCCTTTCGGCGGTGAAATTATCGATGAGCGTGGTGGTT
ATGCCGATCGCGTCACACTACGTCTGAACGTCGAAAACCCGAAACTGTGGAGCGCCGAAATCCCGAATCT
CTATCGTGCGGTGGTTGAACTGCACACCGCCGACGGCACGCTGATTGAAGCAGAAGCCTGCGATGTCGGT
TTCCGCGAGGTGCGGATTGAAAATGGTCTGCTGCTGCTGAACGGCAAGCCGTTGCTGATTCGAGGCGTTA
ACCGTCACGAGCATCATCCTCTGCATGGTCAGGTCATGGATGAGCAGACGATGGTGCAGGATATCCTGCT
GATGAAGCAGAACAACTTTAACGCCGTGCGCTGTTCGCATTATCCGAACCATCCGCTGTGGTACACGCTG
TGCGACCGCTACGGCCTGTATGTGGTGGATGAAGCCAATATTGAAACCCACGGCATGGTGCCAATGAATC
GTCTGACCGATGATCCGCGCTGGCTACCGGCGATGAGCGAACGCGTAACGCGAATGGTGCAGCGCGATCG
TAATCACCCGAGTGTGATCATCTGGTCGCTGGGGAATGAATCAGGCCACGGCGCTAATCACGACGCGCTG
TATCGCTGGATCAAATCTGTCGATCCTTCCCGCCCGGTGCAGTATGAAGGCGGCGGAGCCGACACCACGG
CCACCGATATTATTTGCCCGATGTACGCGCGCGTGGATGAAGACCAGCCCTTCCCGGCTGTGCCGAAATG
GTCCATCAAAAAATGGCTTTCGCTACCTGGAGAGACGCGCCCGCTGATCCTTTGCGAATACGCCCACGCG
ATGGGTAACAGTCTTGGCGGTTTCGCTAAATACTGGCAGGCGTTTCGTCAGTATCCCCGTTTACAGGGCG
GCTTCGTCTGGGACTGGGTGGATCAGTCGCTGATTAAATATGATGAAAACGGCAACCCGTGGTCGGCTTA
CGGCGGTGATTTTGGCGATACGCCGAACGATCGCCAGTTCTGTATGAACGGTCTGGTCTTTGCCGACCGC
ACGCCGCATCCAGCGCTGACGGAAGCAAAACACCAGCAGCAGTTTTTCCAGTTCCGTTTATCCGGGCAAA
CCATCGAAGTGACCAGCGAATACCTGTTCCGTCATAGCGATAACGAGCTCCTGCACTGGATGGTGGCGCT
GGATGGTAAGCCGCTGGCAAGCGGTGAAGTGCCTCTGGATGTCGCTCCACAAGGTAAACAGTTGATTGAA
CTGCCTGAACTACCGCAGCCGGAGAGCGCCGGGCAACTCTGGCTCACAGTACGCGTAGTGCAACCGAACG
CGACCGCATGGTCAGAAGCCGGGCACATCAGCGCCTGGCAGCAGTGGCGTCTGGCGGAAAACCTCAGTGT
GACGCTCCCCGCCGCGTCCCACGCCATCCCGCATCTGACCACCAGCGAAATGGATTTTTGCATCGAGCTG
```

GGTAATAAGCGTTGGCAATTTAACCGCCAGTCAGGCTTTCTTTCACAGATGTGGATTGGCGATAAAAAAC
AACTGCTGACGCCGCTGCGCGATCAGTTCACCCGTGCACCGCTGGATAACGACATTGGCGTAAGTGAAGC
GACCCGCATTGACCCTAACGCCTGGGTCGAACGCTGGAAGGCGGCGGGCCATTACCAGGCCGAAGCAGCG
TTGTTGCAGTGCACGGCAGATACACTTGCTGATGCGGTGCTGATTACGACCGCTCACGCGTGGCAGCATC
AGGGGAAAACCTTATTTATCAGCCGGAAAACCTACCGGATTGATGGTAGTGGTCAAATGGCGATTACCGT
TGATGTTGAAGTGGCGAGCGATACACCGCATCCGGCGCGGATTGGCCTGAACTGCCAGCTGGCGCAGGTA
GCAGAGCGGGTAAACTGGCTCGGATTAGGGCCGCAAGAAAACTATCCCGACCGCCTTACTGCCGCCTGTT
TTGACCGCTGGGATCTGCCATTGTCAGACATGTATACCCCGTACGTCTTCCCGAGCGAAAACGGTCTGCG
CTGCGGGACGCGCGAATTGAATTATGGCCCACACCAGTGGCGCGGCGACTTCCAGTTCAACATCAGCCGC
TACAGTCAACAGCAACTGATGGAAACCAGCCATCGCCATCTGCTGCACGCGGAAGAAGGCACATGGCTGA
ATATCGACGGTTTCCATATGGGGATTGGTGGCGACGACTCCTGGAGCCCGTCAGTATCGGCGGAATTCCA
GCTGAGCGCCGGTCGCTACCATTACCAGTTGGTCTGGTGTCAAAAATAAtaataaccgggcaggccatgt
ctgccgtcgagAAACAGATAGGCCCTCTTCGGAGGGCCTATCTGTTTTTTTTTgtcgacgcgaagcttcc
acagcaagcgaaccggaattgccagctggggcgccctctggtaaggttgggaagccctgcaaagtaaact
ggatggctttcttgccgccaaggatctgatggcgcaggggatcaagatctgatcaagagacaggatgagg
atcgtttcgcATGATTGAACAAGATGGATTGCACGCAGGTTCTCCGGCCGCTTGGGTGGAGAGGCTATTC
GGCTATGACTGGGCACAACAGACAATCGGCTGCTCTGATGCCGCCGTGTTCCGGCTGTCAGCGCAGGGGC
GCCCGGTTCTTTTTGTCAAGACCGACCTGTCCGGTGCCCTGAATGAACTGCAGGACGAGGCAGCGCGGCT
ATCGTGGCTGGCCACGACGGGCGTTCCTTGCGCAGCTGTGCTCGACGTTGTCACTGAAGCGGGAAGGGAC
TGGCTGCTATTGGGCGAAGTGCCGGGGCAGGATCTCCTGTCATCTCACCTTGCTCCTGCCGAGAAAGTAT
CCATCATGGCTGATGCAATGCGGCGGCTGCATACGCTTGATCCGGCTACCTGCCCATTCGACCACCAAGC
GAAACATCGCATCGAGCGAGCACGTACTCGGATGGAAGCCGGTCTTGTCGATCAGGATGATCTGGACGAA
GAGCATCAGGGGCTCGCGCCAGCCGAACTGTTCGCCAGGCTCAAGGCGCGCATGCCCGACGGCGAGGATC
TCGTCGTGACCCATGGCGATGCCTGCTTGCCGAATATCATGGTGGAAAATGGCCGCTTTTCTGGATTCAT
CGACTGTGGCCGGCTGGGTGTGGCGGACCGCTATCAGGACATAGCGTTGGCTACCCGTGATATTGCTGAA
GAGCTTGGCGGCGAATGGGCTGACCGCTTCCTCGTGCTTTACGGTATCGCCGCTCCCGATTCGCAGCGCA
TCGCCTTCTATCGCCTTCTTGACGAGTTCTTCTGAcccggtacctcagcgctagcggagtgtatactggc
ttactatgttggcactgatgagggtgtcagtgaagtgcttcatgtggcaggagaaaaaaggctgcaccgg
tgcgtcagcagaatatgtgatacaggatatattccgcttcctcgctcactgactcgctacgctcggtcgt
tcgactgcggcgagcggaaatggcttacgaacggggcggagatttcctggaagatgccaggaagatactt
aacagggaagtgagagggccgcggcaaagccgtttttccataggctccgcccccctgacaagcatcacga
aatctgacgctcaaatcagtggtggcgaaacccgacaggactataaagataccaggcgtttccccctggc
ggctccctcgtgcgctctcctgttcctgcctttcggtttaccggtgtcattccgctgttatggccgcgtt
tgtctcattccacgcctgacactcagttccgggtaggcagttcgctccaagctggactgtatgcacgaac
cccccgttcagtccgaccgctgcgccttatccggtaactatcgtcttgagtccaacccggaaagacatgc
aaaagcaccactggcagcagccactggtaattgatttagaggagttagtcttgaagtcatgcgccggtta
aggctaaactgaaaggacaagttttggtgactgcgctcctccaagccagttacctcggttcaaagagttg
gtagctcagagaaccttcgaaaaaccgccctgcaaggcggttttttcgttttcagagcaagagattacgc
gcagaccaaaacgatctcaagaagatcatcttattaaggggtctgacgctcagtggaacgaaaactcacg
ttaagggattttggtcatgagattatcaaaaaggatcttcacctagatccttttaaattaaaaatgaagt
tttaaatcaatctaaagtatatatgagtaaacttggtctgacagttaccaatgcttaatcagactagagc
ttccatccgcttgccctcatctgttacgccggcggtag**CCGGCCAGCCTCGCAGAGCAGGATTCCCGTTG**
AGCACCGCCAGGTGCGAATAAGGGACAGTGAAGAAGGAACACCCGCTCGCGGGTGGGCCTACTTCACCTA
TCCTGCCCggctgacgccgttggatacaccaaggaaagtctacacgaaccctttggcaaaatcctgtata
tcgtgcgaattgatccaccgtgcggctgcatgaaatcctggccggtttgtctgatgccaagctggcggcc
tggccggccagcttggccgctgaagaaaccgagcgccgccgtctaaaaaggtgatgtgtatttgagtaaa
acagcttgcgtcatgcggtcgctgcgtatatgatgcgatgagtaaataaacaaatacgcaaggggaacgc

ATGAAGGTTATCGCTGTACTTAACCAGAAAGGCGGGTCAGGCAAGACGACCATCGCAACCCATCTAGCCC
GCGCCCTGCAACTCGCCGGGGCCGATGTTCTGTTAGTCGATTCCGATCCCCAGGGCAGTGCCCGCGATTG
GGCGGCCGTGCGGGAAGATCAACCGCTAACCGTTGTCGGCATCGACCGCCCGACGATTGACCGCGACGTG
AAGGCCATCGGCCGGCGCGACTTCGTAGTGATCGACGGAGCGCCCCAGGCGGCGGACTTGGCTGTGTCCG
CGATCAAGGCAGCCGACTTCGTGCTGATTCCGGTGCAGCCAAGCCCTTACGACATATGGGCCACCGCCGA
CCTGGTGGAGCTGGTTAAGCAGCGCATTGAGGTCACGGATGGAAGGCTACAAGCGGCCTTTGTCGTGTCG
CGGGCGATCAAAGGCACGCGCATCGGCGGTGAGGTTGCCGAGGCGCTGGCCGGGTACGAGCTGCCCATTC
TTGAGTCCCGTATCACGCAGCGCGTGAGCTACCCAGGCACTGCCGCCGCCGGCACAACCGTTCTTGAATC
AGAACCCGAGGGCGACGCTGCCCGCGAGGTCCAGGCGCTGGCCGCTGAAATTAAATCAAAACTCATTTGA
GTTAATGAGGTAAAGAGAAAATGAGCAAAAGCACAAACACGCTAAGTGCCGGCCGTCCGAGCGCACGCAG
CAGCAAGGCTGCAACGTTGGCCAGCCTGGCAGACACGCCAGCCATGAAGCGGGTCAACTTTCAGTTGCCG
GCGGAGGATCACACCAAGCTGAAGATGTACGCGGTACGCCAAGGCAAGACCATTACCGAGCTGCTATCTG
AATACATCGCGCAGCTACCAGAGTAAATGAGCAAATGAATAAATGAGTAGATGAATTTTAGCGGCTAAAG
GAGGCGGCATGGAAAATCAAGAACAACCAGGCACCGACGCCGTGGAATGCCCCATGTGTGGAGGAACGGG
CGGTTGGCCAGGCGTAAGCGGCTGGGTTGTCTGCCGGCCCTGCAATGGCACTGGAACCCCCAAGCCCGAG
GAATCGGCGTGACGGTCGCAAACCATCCGGCCCGGTACAAATCGGCGCGGCGCTGGGTGATGACCTGGTG
GAGAAGTTGAAGGCCGCGCAGGCCGCCCAGCGGCAACGCATCGAGGCAGAAGCACGCCCCGGTGAATCGT
GGCAAGCGGCCGCTGATCGAATCCGCAAAGAATCCCGGCAACCGCCGGCAGCCGGTGCGCCGTCGATTAG
GAAGCCGCCCAAGGGCGACGAGCAACCAGATTTTTTCGTTCCGATGCTCTATGACGTGGGCACCCGCGAT
AGTCGCAGCATCATGGACGTGGCCGTTTTCCGTCTGTCGAAGCGTGACCGACGAGCTGGCGAGGTGATCC
GCTACGAGCTTCCAGACGGGCACGTAGAGGTTTCCGCAGGGCCGGCCGGCATGGCCAGTGTGTGGGATTA
CGACCTGGTACTGATGGCGGTTTCCCATCTAACCGAATCCATGAACCGATACCGGGAAGGGAAGGGAGAC
AAGCCCGGCCGCGTGTTCCGTCCACACGTTGCGGACGTACTCAAGTTCTGCCGGCGAGCCGATGGCGGAA
AGCAGAAAGACGACCTGGTAGAAACCTGCATTCGGTTAAACACCACGCACGTTGCCATGCAGCGTACGAA
GAAGGCCAAGAACGGCCGCCTGGTGACGGTATCCGAGGGTGAAGCCTTGATTAGCCGCTACAAGATCGTA
AAGAGCGAAACCGGGCGGCCGGAGTACATCGAGATCGAGCTAGCTGATTGGATGTACCGCGAGATCACAG
AAGGCAAGAACCCGGACGTGCTGACGGTTCACCCCGATTACTTTTTGATCGATCCCGGCATCGGCCGTTT
TCTCTACCGCCTGGCACGCCGCGCCGCAGGCAAGGCAGAAGCCAGATGGTTGTTCAAGACGATCTACGAA
CGCAGTGGCAGCGCCGGAGAGTTCAAGAAGTTCTGTTTCACCGTGCGCAAGCTGATCGGGTCAAATGACC
TGCCGGAGTACGATTTGAAGGAGGAGGCGGGGCAGGCTGGCCCGATCCTAGTCATGCGCTACCGCAACCT
GATCGAGGGCGAAGCATCCGCCGGTTCCTAATGTACGGAGCAGATGCTAGGGCAAATTGCCCTAGCAGGG
GAAAAAGGTCGAAAAGGTCTCTTTCCTGTGGATAGCACGTACATTGGGAACCCAAAGCCGTACATTGGGA
ACCGGAACCCGTACATTGGGAACCCAAAGCCGTACATTGGGAACCGGTCACACATGTAAGTGACTGATAT
AAAAGAGAAAAAAGGCGATTTTTCCGCCTAAAACTCTTTAAAACTTATTAAAACTCTTAAAACCCGCCTG
GCCTGTGCATAactgtctggccagcgcacagccgaagagctgcaaaaagcgcctacccttcggtcgctgc
gctccctacgccccgccgcttcgcgtcggcctatcgcggccgctggccgctcaaaaatggctggcctacg
gccaggcaatctaccagggcgcggacaagccgcgccgtcgccactcgaccgccggcgcccacatcaaggc
accctgcct

pKJ140 (8500 bp)
Vector used for *lacZ* reporter gene studies; *nreA*$^{+}$
BamHI: 28-33, XbaI: 37-42 (for promoter/operator cloning)
RBS1: 40-54 bp
lacZ (*ampR*): 55-3129 bp
T7hyb1 Terminator: 3162-3203 bp
Kanamycin/Neomycin resistance (*kanR*): 3371-4165 bp
KpnI: 4169-4174
RBS2: 4175-4187 bp
***nreA*: 4188-4469 bp** (absent in pKJ138)
KpnI: 4470-4475
p15A *ori*: 4746-5291 bp
RK2 *oriT*: 5520-5629 bp (for conjugation)
pVS1 *ori/sta*: 5902-8292 bp (for *Agrobacterium* replication)

gagctcaccgaattcccactcgagccaGGATCCaccTCTAGAAGGAGGTTACATATGACCATGATTACGG
ATTCACTGGCCGTCGTTTTACAACGTCGTGACTGGGAAAACCCTGGCGTTACCCAACTTAATCGCCTTGC
AGCACATCCCCCTTTCGCCAGCTGGCGTAATAGCGAAGAGGCCCGCACCGATCGCCCTTCCCAACAGTTG
CGCAGCCTGAATGGCGAATGGCGCTTTGCCTGGTTTCCGGCACCAGAAGCGGTGCCGGAAAGCTGGCTGG
AGTGCGATCTTCCTGAGGCCGATACTGTCGTCGTCCCCTCAAACTGGCAGATGCACGGTTACGATGCGCC
CATCTACACCAACGTGACCTATCCCATTACGGTCAATCCGCCGTTTGTTCCCACGGAGAATCCGACGGGT
TGTTACTCGCTCACATTTAATGTTGATGAAAGCTGGCTACAGGAAGGCCAGACGCGAATTATTTTTGATG
GCGTTAACTCGGCGTTTCATCTGTGGTGCAACGGGCGCTGGGTCGGTTACGGCCAGGACAGTCGTTTGCC
GTCTGAATTTGACCTGAGCGCATTTTTACGCGCCGGAGAAAACCGCCTCGCGGTGATGGTGCTGCGCTGG
AGTGACGGCAGTTATCTGGAAGATCAGGATATGTGGCGGATGAGCGGCATTTTCCGTGACGTCTCGTTGC
TGCATAAACCGACTACACAAATCAGCGATTTCCATGTTGCCACTCGCTTTAATGATGATTTCAGCCGCGC
TGTACTGGAGGCTGAAGTTCAGATGTGCGGCGAGTTGCGTGACTACCTACGGGTAACAGTTTCTTTATGG
CAGGGTGAAACGCAGGTCGCCAGCGGCACCGCGCCTTTCGGCGGTGAAATTATCGATGAGCGTGGTGGTT
ATGCCGATCGCGTCACACTACGTCTGAACGTCGAAAACCCGAAACTGTGGAGCGCCGAAATCCCGAATCT
CTATCGTGCGGTGGTTGAACTGCACACCGCCGACGGCACGCTGATTGAAGCAGAAGCCTGCGATGTCGGT
TTCCGCGAGGTGCGGATTGAAAATGGTCTGCTGCTGCTGAACGGCAAGCCGTTGCTGATTCGAGGCGTTA
ACCGTCACGAGCATCATCCTCTGCATGGTCAGGTCATGGATGAGCAGACGATGGTGCAGGATATCCTGCT
GATGAAGCAGAACAACTTTAACGCCGTGCGCTGTTCGCATTATCCGAACCATCCGCTGTGGTACACGCTG
TGCGACCGCTACGGCCTGTATGTGGTGGATGAAGCCAATATTGAAACCCACGGCATGGTGCCAATGAATC
GTCTGACCGATGATCCGCGCTGGCTACCGGCGATGAGCGAACGCGTAACGCGAATGGTGCAGCGCGATCG
TAATCACCCGAGTGTGATCATCTGGTCGCTGGGGAATGAATCAGGCCACGGCGCTAATCACGACGCGCTG
TATCGCTGGATCAAATCTGTCGATCCTTCCCGCCCGGTGCAGTATGAAGGCGGCGGAGCCGACACCACGG
CCACCGATATTATTTGCCCGATGTACGCGCGCGTGGATGAAGACCAGCCCTTCCCGGCTGTGCCGAAATG
GTCCATCAAAAAATGGCTTTCGCTACCTGGAGAGACGCGCCCGCTGATCCTTTGCGAATACGCCCACGCG
ATGGGTAACAGTCTTGGCGGTTTCGCTAAATACTGGCAGGCGTTTCGTCAGTATCCCCGTTTACAGGGCG
GCTTCGTCTGGGACTGGGTGGATCAGTCGCTGATTAAATATGATGAAAACGGCAACCCGTGGTCGGCTTA
CGGCGGTGATTTTGGCGATACGCCGAACGATCGCCAGTTCTGTATGAACGGTCTGGTCTTTGCCGACCGC
ACGCCGCATCCAGCGCTGACGGAAGCAAAACACCAGCAGCAGTTTTTCCAGTTCCGTTTATCCGGGCAAA
CCATCGAAGTGACCAGCGAATACCTGTTCCGTCATAGCGATAACGAGCTCCTGCACTGGATGGTGGCGCT
GGATGGTAAGCCGCTGGCAAGCGGTGAAGTGCCTCTGGATGTCGCTCCACAAGGTAAACAGTTGATTGAA
CTGCCTGAACTACCGCAGCCGGAGAGCGCCGGGCAACTCTGGCTCACAGTACGCGTAGTGCAACCGAACG
CGACCGCATGGTCAGAAGCCGGGCACATCAGCGCCTGGCAGCAGTGGCGTCTGGCGGAAAACCTCAGTGT

GACGCTCCCCGCCGCGTCCCACGCCATCCCGCATCTGACCACCAGCGAAATGGATTTTTGCATCGAGCTG
GGTAATAAGCGTTGGCAATTTAACCGCCAGTCAGGCTTTCTTTCACAGATGTGGATTGGCGATAAAAAAC
AACTGCTGACGCCGCTGCGCGATCAGTTCACCCGTGCACCGCTGGATAACGACATTGGCGTAAGTGAAGC
GACCCGCATTGACCCTAACGCCTGGGTCGAACGCTGGAAGGCGGCGGGCCATTACCAGGCCGAAGCAGCG
TTGTTGCAGTGCACGGCAGATACACTTGCTGATGCGGTGCTGATTACGACCGCTCACGCGTGGCAGCATC
AGGGGAAAACCTTATTTATCAGCCGGAAAACCTACCGGATTGATGGTAGTGGTCAAATGGCGATTACCGT
TGATGTTGAAGTGGCGAGCGATACACCGCATCCGGCGCGGATTGGCCTGAACTGCCAGCTGGCGCAGGTA
GCAGAGCGGGTAAACTGGCTCGGATTAGGGCCGCAAGAAAACTATCCCGACCGCCTTACTGCCGCCTGTT
TTGACCGCTGGGATCTGCCATTGTCAGACATGTATACCCCGTACGTCTTCCCGAGCGAAAACGGTCTGCG
CTGCGGGACGCGCGAATTGAATTATGGCCCACACCAGTGGCGCGGCGACTTCCAGTTCAACATCAGCCGC
TACAGTCAACAGCAACTGATGGAAACCAGCCATCGCCATCTGCTGCACGCGGAAGAAGGCACATGGCTGA
ATATCGACGGTTTCCATATGGGGATTGGTGGCGACGACTCCTGGAGCCCGTCAGTATCGGCGGAATTCCA
GCTGAGCGCCGGTCGCTACCATTACCAGTTGGTCTGGTGTCAAAAATAAtaataaccgggcaggccatgt
ctgccgtcgagAAACAGATAGGCCCTCTTCGGAGGGCCTATCTGTTTTTTTTTgtcgacgcgaagcttcc
acagcaagcgaaccggaattgccagctggggcgccctctggtaaggttgggaagccctgcaaagtaaact
ggatggctttcttgccgccaaggatctgatggcgcaggggatcaagatctgatcaagagacaggatgagg
atcgtttcgcATGATTGAACAAGATGGATTGCACGCAGGTTCTCCGGCCGCTTGGGTGGAGAGGCTATTC
GGCTATGACTGGGCACAACAGACAATCGGCTGCTCTGATGCCGCCGTGTTCCGGCTGTCAGCGCAGGGGC
GCCCGGTTCTTTTTGTCAAGACCGACCTGTCCGGTGCCCTGAATGAACTGCAGGACGAGGCAGCGCGGCT
ATCGTGGCTGGCCACGACGGGCGTTCCTTGCGCAGCTGTGCTCGACGTTGTCACTGAAGCGGGAAGGGAC
TGGCTGCTATTGGGCGAAGTGCCGGGGCAGGATCTCCTGTCATCTCACCTTGCTCCTGCCGAGAAAGTAT
CCATCATGGCTGATGCAATGCGGCGGCTGCATACGCTTGATCCGGCTACCTGCCCATTCGACCACCAAGC
GAAACATCGCATCGAGCGAGCACGTACTCGGATGGAAGCCGGTCTTGTCGATCAGGATGATCTGGACGAA
GAGCATCAGGGGCTCGCGCCAGCCGAACTGTTCGCCAGGCTCAAGGCGCGCATGCCCGACGGCGAGGATC
TCGTCGTGACCCATGGCGATGCCTGCTTGCCGAATATCATGGTGGAAAATGGCCGCTTTTCTGGATTCAT
CGACTGTGGCCGGCTGGGTGTGGCGGACCGCTATCAGGACATAGCGTTGGCTACCCGTGATATTGCTGAA
GAGCTTGGCGGCGAATGGGCTGACCGCTTCCTCGTGCTTTACGGTATCGCCGCTCCCGATTCGCAGCGCA
TCGCCTTCTATCGCCTTCTTGACGAGTTCTTCTGAcccGGTACCAGGAGGTATACAT**ATGAATGAGCGTC**
CCCACGTCCACGAAACCCATCCTGCCATCATCAAGCGGCTGAAGCGCGCCGACGGCCATCTGCGCGGGAT
CGTCGAGATGATCGAGGCCGGGCGACCGTGCCTCGACATCGCCCAGCAACTTCATGCCGTGGAGAAGGCC
ATCGCCCAGGCCAAGAAGACGCTGATCCAGGACCACCTCAACCACTGCCTTGAAGACGTCGTCGGACCGC
TGGCGCTCGAGCAGCGCCGCTCGATCGACGAGTTCAAGGATATCACCAAGTACCTGTGAGGTACCtcagc
gctagcggagtgtatactggcttactatgttggcactgatgagggtgtcagtgaagtgcttcatgtggca
ggagaaaaaaggctgcaccggtgcgtcagcagaatatgtgatacaggatatattccgcttcctcgctcac
tgactcgctacgctcggtcgttcgactgcggcgagcggaaatggcttacgaacggggcggagatttcctg
gaagatgccaggaagatacttaacagggaagtgagagggccgcggcaaagccgtttttccataggctccg
cccccctgacaagcatcacgaaatctgacgctcaaatcagtggtggcgaaacccgacaggactataaaga
taccaggcgtttccccctggcggctccctcgtgcgctctcctgttcctgcctttcggtttaccggtgtca
ttccgctgttatggccgcgtttgtctcattccacgcctgacactcagttccgggtaggcagttcgctcca
agctggactgtatgcacgaaccccccgttcagtccgaccgctgcgccttatccggtaactatcgtcttga
gtccaacccggaaagacatgcaaaagcaccactggcagcagccactggtaattgatttagaggagttagt
cttgaagtcatgcgccggttaaggctaaactgaaaggacaagttttggtgactgcgctcctccaagccag
ttacctcggttcaaagagttggtagctcagagaaccttcgaaaaaccgccctgcaaggcggttttttcgt
tttcagagcaagagattacgcgcagaccaaaacgatctcaagaagatcatcttattaaggggtctgacgc
tcagtggaacgaaaactcacgttaagggattttggtcatgagattatcaaaaaggatcttcacctagatc
cttttaaattaaaaatgaagttttaaatcaatctaaagtatatatgagtaaacttggtctgacagttacc
aatgcttaatcagactagagcttccatccgcttgccctcatctgttacgccggcggtag**CCGGCCAGCCT**

CGCAGAGCAGGATTCCCGTTGAGCACCGCCAGGTGCGAATAAGGGACAGTGAAGAAGGAACACCCGCTCG
CGGGTGGGCCTACTTCACCTATCCTGCCCggctgacgccgttggatacaccaaggaaagtctacacgaac
cctttggcaaaatcctgtatatcgtgcgaattgatccaccgtgcggctgcatgaaatcctggccggtttg
tctgatgccaagctggcggcctggccggccagcttggccgctgaagaaaccgagcgccgccgtctaaaaa
ggtgatgtgtatttgagtaaaacagcttgcgtcatgcggtcgctgcgtatatgatgcgatgagtaaataa
acaaatacgcaaggggaacgcATGAAGGTTATCGCTGTACTTAACCAGAAAGGCGGGTCAGGCAAGACGA
CCATCGCAACCCATCTAGCCCGCGCCCTGCAACTCGCCGGGGCCGATGTTCTGTTAGTCGATTCCGATCC
CCAGGGCAGTGCCCGCGATTGGGCGGCCGTGCGGGAAGATCAACCGCTAACCGTTGTCGGCATCGACCGC
CCGACGATTGACCGCGACGTGAAGGCCATCGGCCGGCGCGACTTCGTAGTGATCGACGGAGCGCCCCAGG
CGGCGGACTTGGCTGTGTCCGCGATCAAGGCAGCCGACTTCGTGCTGATTCCGGTGCAGCCAAGCCCTTA
CGACATATGGGCCACCGCCGACCTGGTGGAGCTGGTTAAGCAGCGCATTGAGGTCACGGATGGAAGGCTA
CAAGCGGCCTTTGTCGTGTCGCGGGCGATCAAAGGCACGCGCATCGGCGGTGAGGTTGCCGAGGCGCTGG
CCGGGTACGAGCTGCCCATTCTTGAGTCCCGTATCACGCAGCGCGTGAGCTACCCAGGCACTGCCGCCGC
CGGCACAACCGTTCTTGAATCAGAACCCGAGGGCGACGCTGCCCGCGAGGTCCAGGCGCTGGCCGCTGAA
ATTAAATCAAAACTCATTTGAGTTAATGAGGTAAAGAGAAAATGAGCAAAAGCACAAACACGCTAAGTGC
CGGCCGTCCGAGCGCACGCAGCAGCAAGGCTGCAACGTTGGCCAGCCTGGCAGACACGCCAGCCATGAAG
CGGGTCAACTTTCAGTTGCCGGCGGAGGATCACACCAAGCTGAAGATGTACGCGGTACGCCAAGGCAAGA
CCATTACCGAGCTGCTATCTGAATACATCGCGCAGCTACCAGAGTAAATGAGCAAATGAATAAATGAGTA
GATGAATTTTAGCGGCTAAAGGAGGCGGCATGGAAAATCAAGAACAACCAGGCACCGACGCCGTGGAATG
CCCCATGTGTGGAGGAACGGGCGGTTGGCCAGGCGTAAGCGGCTGGGTTGTCTGCCGGCCCTGCAATGGC
ACTGGAACCCCCAAGCCCGAGGAATCGGCGTGACGGTCGCAAACCATCCGGCCCGGTACAAATCGGCGCG
GCGCTGGGTGATGACCTGGTGGAGAAGTTGAAGGCCGCGCAGGCCGCCCAGCGGCAACGCATCGAGGCAG
AAGCACGCCCCGGTGAATCGTGGCAAGCGGCCGCTGATCGAATCCGCAAAGAATCCCGGCAACCGCCGGC
AGCCGGTGCGCCGTCGATTAGGAAGCCGCCCAAGGGCGACGAGCAACCAGATTTTTTCGTTCCGATGCTC
TATGACGTGGGCACCCGCGATAGTCGCAGCATCATGGACGTGGCCGTTTTCCGTCTGTCGAAGCGTGACC
GACGAGCTGGCGAGGTGATCCGCTACGAGCTTCCAGACGGGCACGTAGAGGTTTCCGCAGGGCCGGCCGG
CATGGCCAGTGTGTGGGATTACGACCTGGTACTGATGGCGGTTTCCCATCTAACCGAATCCATGAACCGA
TACCGGGAAGGGAAGGGAGACAAGCCCGGCCGCGTGTTCCGTCCACACGTTGCGGACGTACTCAAGTTCT
GCCGGCGAGCCGATGGCGGAAAGCAGAAAGACGACCTGGTAGAAACCTGCATTCGGTTAAACACCACGCA
CGTTGCCATGCAGCGTACGAAGAAGGCCAAGAACGGCCGCCTGGTGACGGTATCCGAGGGTGAAGCCTTG
ATTAGCCGCTACAAGATCGTAAAGAGCGAAACCGGGCGGCCGGAGTACATCGAGATCGAGCTAGCTGATT
GGATGTACCGCGAGATCACAGAAGGCAAGAACCCGGACGTGCTGACGGTTCACCCCGATTACTTTTTGAT
CGATCCCGGCATCGGCCGTTTTCTCTACCGCCTGGCACGCCGCGCCGCAGGCAAGGCAGAAGCCAGATGG
TTGTTCAAGACGATCTACGAACGCAGTGGCAGCGCCGGAGAGTTCAAGAAGTTCTGTTTCACCGTGCGCA
AGCTGATCGGGTCAAATGACCTGCCGGAGTACGATTTGAAGGAGGAGGCGGGGCAGGCTGGCCCGATCCT
AGTCATGCGCTACCGCAACCTGATCGAGGGCGAAGCATCCGCCGGTTCCTAATGTACGGAGCAGATGCTA
GGGCAAATTGCCCTAGCAGGGGAAAAAGGTCGAAAAGGTCTCTTTCCTGTGGATAGCACGTACATTGGGA
ACCCAAAGCCGTACATTGGGAACCGGAACCCGTACATTGGGAACCCAAAGCCGTACATTGGGAACCGGTC
ACACATGTAAGTGACTGATATAAAAGAGAAAAAAGGCGATTTTTCCGCCTAAAACTCTTTAAAACTTATT
AAAACTCTTAAAACCCGCCTGGCCTGTGCATAactgtctggccagcgcacagccgaagagctgcaaaaag
cgcctacccttcggtcgctgcgctccctacgccccgccgcttcgcgtcggcctatcgcggccgctggccg
ctcaaaaatggctggcctacggccaggcaatctaccagggcgcggacaagccgcgccgtcgccactcgac
cgccggcgcccacatcaaggcaccctgcct

pKJ213 (1790 bp)
Intermediate vector used for *nreA* mutagenesis prior to *nreA* transfer to pKJ227
pKan promoter: 42-70 bp
Chloramphenicol resistance (CmR): 101-660 bp
RBS: 776-788 bp
nreA: 789-1070 bp
Terminator: 1098-1126
ColE1 *ori*: 1195-1783 bp

gcaacgcggcctttttacggttcctggccttttgagctcaaTTGCCAGCTGGAGCGCCCTCTGGTAAGGT
tgggaagtctagactttaggaggtatacatATGGAGAAAAAAATCACTGGATATACCACCGTTGATATAT
CCCAATGGCATCGTAAAGAACATTTTGAGGCATTTCAGTCAGTTGCTCAATGTACCTATAACCAGACCGT
TCAGCTGGATATTACGGCCTTTTTAAAGACCGTAAAGAAAAATAAGCACAAGTTTTATCCGGCCTTTATT
CACATTCTTGCCCGCCTGATGAATGCTCATCCGGAATTCCGTATGGCAATGAAAGACGGTGAGCTGGTGA
TATGGGATAGTGTTCACCCTTGTTACACCGTTTTCCATGAGCAAACTGAAACGTTTTCATCGCTCTGGAG
TGAATACCACGACGATTTCCGGCAGTTTCTACACATATATTCGCAAGATGTGGCGTGTTACGGTGAAAAC
CTGGCCTATTTCCCTAAAGGGTTTATTGAGAATATGTTTTTCGTCTCAGCCAATCCCTGGGTGAGTTTCA
CCAGTTTTGATTTAAACGTGGCCAATATGGACAACTTCTTCGCCCCCGTTTTCACCATGGGCAAATATTA
TACGCAAGGCGACAAGGTGCTGATGCCGCTGGCGATTCAGGTTCATCATGCCGTTTGTGATGGCTTCCAT
GTCGGCAGAATGCTTAATGAATTACAACAGTACTGCGATGAGTGGCAGGGTGGTGCGTAActcgagcgtg
gtaccAGGAGGTATACATATGAATGAGCGTCCCCACGTCCACGAAACCCATCCTGCCATCATCAAGCGGC
TGAAGCGCGCCGACGGCCATCTGCGCGGATCGTCGAGATGATCGAGGCCGGGCGACCGTGCCTCGACAT
CGCCCAGCAACTTCATGCCGTGGAGAAGGCCATCGCCCAGGCCAAGAAGACGCTGATCCAGGACCACCTC
AACCACTGCCTTGAAGACGTCGTCGGACCGCTGGCGCTCGAGCAGCGCCGCTCGATCGACGAGTTCAAGG
ATATCACCAAGTACCTGTGAggtaagcttaggctagctgacacgctt**AAGGCCTCCCAATCGGGAGGCCT**
TTTTTTgaccgattaccctgttatccctagttccactgagcgtcaggatccgtagaaaagatcaaaggat
cttcTTGAGATCCTTTTTTTCTGCGCGTAATCTGCTGCTTGCAAACAAAAAAACCACCGCTACCAGCGGT
GGTTTGTTTGCCGGATCAAGAGCTACCAACTCTTTTTCCGAAGGTAACTGGCTTCAGCAGAGCGCAGATA
CCAAATACTGTCCTTCTAGTGTAGCCGTAGTTAGGCCACCACTTCAAGAACTCTGTAGCACCGCCTACAT
ACCTCGCTCTGCTAATCCTGTTACCAGTGGCTGCTGCCAGTGGCGATAAGTCGTGTCTTACCGGGTTGGA
CTCAAGACGATAGTTACCGGATAAGGCGCAGCGGTCGGGCTGAACGGGGGGTTCGTGCACACAGCCCAGC
TTGGAGCGAACGACCTACACCGAACTGAGATACCTACAGCGTGAGCTATGAGAAAGCGCCACGCTTCCCG
AAGGGAGAAAGGCGGACAGGTATCCGGTAAGCGGCAGGGTCGGAACAGGAGAGCGCACGAGGGAGCTTCC
AGGGGGAAACGCCTGGTATCTTTATAGTCCTGTCGGGTTTCGCCACCTCTGACTTGAGCGTCGATTTTTG
TGATGCTCGTCAGGGGGGCGGAGCCTATGGAAaaacgcca
